# MiR-181a protects the heart against myocardial infarction by regulating mitochondrial fission via targeting programmed cell death protein 4

**DOI:** 10.1038/s41598-024-57206-8

**Published:** 2024-03-19

**Authors:** Jianbing Zhu, Qian Wang, Zeqi Zheng, Leilei Ma, Junjie Guo, Hongtao Shi, Ru Ying, Beilei Gao, Shanshan Chen, Siyang Yu, Bin Yuan, Xiaoping Peng, Junbo Ge

**Affiliations:** 1 Department of Cardiology, The First Affiliated Hospital of Nanchang University, Nanchang, China; 2 Jiangxi Hypertension Research Institute, Nanchang, China; 3 Department of Blood Transfusion, The First Affiliated Hospital of Nanchang University, Nanchang, China; 4 Department of Cardiology, Shanghai Institute of Cardiovascular Diseases, Zhongshan Hospital, Fudan University, Shanghai, China; 5 Department of Cardiology, Affiliated Hospital of Qingdao University, Qingdao, China; 6 Department of Cardiovascular Surgery, The First Affiliated Hospital of Nanchang University, Nanchang, China

**Keywords:** Myocardial infarction (MI), Mitochondrial fission, miR-181a, Programmed cell death protein 4 (PDCD4), Bid, p53, Apoptosis, Mitochondria, miRNAs, Cardiomyopathies

## Abstract

Worldwide, myocardial infarction (MI) is the leading cause of death and disability-adjusted life years lost. Recent researches explored new methods of detecting biomarkers that can predict the risk of developing myocardial infarction, which includes identifying genetic markers associated with increased risk. We induced myocardial infarction in mice by occluding the left anterior descending coronary artery and performed TTC staining to assess cell death. Next, we performed ChIP assays to measure the enrichment of histone modifications at the promoter regions of key genes involved in mitochondrial fission. We used qPCR and western blot to measure expression levels of relative apoptotic indicators. We report that miR-181a inhibits myocardial ischemia-induced apoptosis and preserves left ventricular function after MI. We show that programmed cell death protein 4 (PDCD4) is the target gene involved in miR-181a-mediated anti-ischemic injury, which enhanced BID recruitment to the mitochondria. In addition, we discovered that p53 inhibits the expression of miR-181a via transcriptional regulation. Here, we discovered for the first time a mitochondrial fission and apoptosis pathway which is controlled by miR-181a and involves PDCD4 and BID. This pathway may be controlled by p53 transcriptionally, and we presume that miR-181a may lead to the discovery of new therapeutic and preventive targets for ischemic heart diseases.

## Introduction

Myocardial infarction (MI), the primary cause of death and loss of years of productive life worldwide, is caused by myocardial ischemia, hypoxia resulting from vascular constriction or blockage due to atherosclerosis of the coronary artery^1^. Previous research has shown that increased apoptosis exacerbates MI and heart failure, while inhibiting apoptosis protects the heart from MI^2^. Apoptosis includes the process of mitochondrial fission as one of its stages. Mitochondria are constantly fusing with one another and breaking apart inside of cells to form a dynamic network. Maintaining proper mitochondrial morphology in human and animal cardiac myocytes requires mitochondrial dynamics, which is also vital for good cardiomyocyte respiratory and contractile function^3^.

MicroRNAs (miRNAs), are small molecules made up of non-coding RNA that have been discovered to have a significant role in the regulation of pathophysiological processes^4^. Several functional investigations have shown that different miRNAs affect MI differently^5,6^. MiRNAs influence myocardial ischemia response differently from pharmaceuticals since they promote pro-survival signaling networks to govern cardiomyocyte survival and cardiac function in animals after a MI^7^. The major roles that miRNAs play in the cardiovascular system have led to new insights into the processes of cardiovascular disease and the identification of potential diagnostic and therapeutic strategies for ischemic heart disease^8^.

Programmed cell death protein 4 (PDCD4) was initially identified as a protein produced by apoptotic stimuli that functions as a tumor suppressor^9^. It has been shown that PDCD4 play a significant part in the pro-apoptotic effect of cardiomyocytes that is generated by ischemia injury, oxidative stress, or lipopolysaccharide^10^. However, it is unclear whether PDCD4 modulates mitochondrial fission to contribute to MI. BID, a proapoptotic BCL-2 family member, is cleaved into the active form, tBid upon Fas receptor stimulation^11^. Following this, tBid accumulates in the outer mitochondrial membrane, which helps to encourage mitochondria fission. Mitochondrial fission is an essential stage in the process of committing a cell to apoptosis by using the effector proteins Bax or Bak^11^.

Several researches have recently demonstrated that ischemia postconditioning-affected miR-181a is implicated in the pathogenesis of MI and attenuates ox-LDL-stimulated immune inflammatory responses^12,13^. In this study, we found that miR-181a can preserve the heart against MI by preventing cardiomyocyte apoptosis through targeting PDCD4-mediated BID activation and consequently participated in mitochondrial fission. Our study indicates a previously unknown mechanism for posttranscriptional regulation of mitochondrial fission pathway for apoptosis involving miR-181a, PDCD4 and BID.

## Methods

### Establishment of MI model

Mice were purchased from Shanghai Experimental Animal Center of Chinese Academic of Sciences (Shanghai, China). All animal procedures were performed in accordance with the National Institutes of Health Guidelines on the Use of Laboratory Animals. The mice were treated and tested according to the Animal Research: Reporting In Vivo Experiments Guidelines (ARRIVE Guidelines). All procedures were approved by the Animal Research Committee of the Zhongshan Hospital, Fudan University. The animals were cared for following the Guiding Principles in the Use and Care of Animals published by the US National Institutes of Health (NIH Publication No. 85-23, revised 1996). All of the manipulations and analyses involving the animals were carried out in a manner that was blind to intervention and genotype. In order to produce MI, permanent coronary artery ligation was performed on mice utilizing a novel and speedy procedure that did not include ventilation described by previous research^14^. Mice were given an inhalational anesthetic (isoflurane, 2%; no ventilation) and placed supine on a heating pad. After the pectoral major and minor muscles were dissected, the fourth intercostal gap was uncovered. Utilizing a mosquito clamp to puncture the pleural membrane and pericardium, the heart was carefully popped out through the orifice. Following the closure of the left anterior descending coronary artery (LAD) with a 6–0 silk suture, the heart was immediately returned back into the intra-thoracic space where it had been before the procedure. The only difference between the procedures performed on MI animals and those performed on sham mice was that the LAD in the sham mice was not ligated. For euthanasia, the mice were anesthetized with isoflurane and then sacrificed through manual cervical dislocation.

### Terminal deoxynucleotidyl transferase (TdT)-mediated dUTP Nick End Labeling (TUNEL) staining

TUNEL staining was conducted using a fluorescence detection kit (Roche) according to the manufacturer's instructions. Nuclear counterstaining was carried out with DAPI. A TUNEL assay was conducted in accordance with the manufacturer's instructions using a Fluorescein (Roche) in situ cell death detection kit. Ten random fields were run for each area to determine the apoptotic rate. using a fluorescence detection kit (Roche) according to the manufacturer’s protocol. DAPI was used for nuclear counterstaining. TUNEL was performed using an In Situ Cell Death Detection kit, Fluorescein (Roche) per the manufacturer’s protocol. We performed ten random fields per section to calculate numbers of apoptosis.

### Measurement of myocardial infarct size

At 24 h post-coronary ligation, mice were euthanized and the hearts were sliced into 2-mm thick sections before being examined. After being fixed in 4% PFA at 4 °C, the heart slices were stained in 1.5% TTC for 30 min at 37 °C to distinguish between the infarcted and non-infarcted zone. The area of non-infarcted was demarcated as a red area (TTC positive), whereas the infarcted area was defined as TTC unstained area (write color). Using the imaging software ImagePro (V9.3, Bio-Rad Laboratories), the infarct area was calculated from the sliced images. Percentage infarct compared to left ventricular (LV) size was determined using planimetry, as previously described^15^.

### Isolation, culture, hypoxia treatment and adenoviral infection of neonatal rat ventricular myocytes

Primary cultures of neonatal rat cardiac ventricular myocytes from 2-d-old Sprague–Dawley rats were performed as described previously^16^. We washed and minced hearts after dissection in HEPES-buffered saline solution containing 130 mM NaCl, 1 mM NaH_2_PO_4_, 3 mM KCl, 4 mM glucose and 20 mM HEPES. The minced tissue and ventricular cells were dispersed by digestion with collagenase type IV (0.45 mg/ml), 0.1% trypsin, and 15 g/ml DNase I. We resuspended cells in DMEM/F-12 medium containing 10% FBS, 1% penicillin, 1% streptomycin. Isolated Cells were preplaced at 37 °C for 2 h, diluted them to 1 × 10^6^ cells and then seeded into six-well plates.

To establish the hypoxia model, we replaced the culture medium with serum-free DMEM and placed the cells in an anaerobic chamber containing 94% N_2_, 5% CO_2_, and 1% O_2_. Cells in the normoxia group were cultured in serum-free DMEM and placed in a normoxic incubator. Unless otherwise indicated, the cells were subjected to hypoxia for 24 h.

To construct adenovirus encoding miR-181a, rat genomic sequence harboring the pre-miR-181a was amplified using the following primer set: 5′-ACGGTACCTGCAGGATCTCAGCAAAGGA-3′, 5′- ACCTCGAGAGGAACAGTGAGCAGTAGGA-3′, and finally cloned into the adenovirus system. The introduction of mutations into pre-miR-181a (WT: 5’-GGTACAATCAACGGTCGATGGTT-3’, mutant: 5’- GGTGTCCTCAACGGTCGATGGTT-3’) was generated using QuikChange II XL Site-Directed Mutagenesis Kit (Stratagene). Neonatal rat ventricular cardiomyocytes (NRVCs) were infected at the indicated moi of adenoviruses amplified in HEK293 cells according to the manufacturer’s instructions.

### Magnetic resonance imaging

MI and sham mice underwent serial assessment of cardiac dimensions and function by a small animal high-resolution magnetic resonance imaging scanner (7.0 T; Bruker, BioSpec 70/20 USR, Germany) before and 4 weeks after MI. Each mouse was administered via an MRI-compatible mobile inhalation anesthesia system (Matrx) under 2% isoflurane. Electrocardiography (ECG) was performed on a small animal monitoring and gating system (Small Animal Instruments, Incorporated) by three ECG leads inserted in mice. Cine short-axis images at 12 or 13 phases per cardiac cycle were obtained by an ECG-triggered two-dimensional gradient echo sequence with a flip angle of 45°, slice thickness 1 mm, repetition time of 200 ms, and echo time of 2.7 ms. All magnetic resonance imaging data were analyzed with Segment (http://segment.heiberg.se). The LV ejection fraction (LVEF), LV fractional shortening (LVFS), diastolic LV internal diameters (LVIDd), systolic LV internal diameters (LVIDs), and systolic volume (SV) were measured.

### Masson’s trichrome staining

Hearts were obtained, fixed in 4% paraformaldehyde, embedded in paraffin, and sections were stained with Masson-Trichrome to examine cardiac fibrosis. Then the cardiac collagen area was assessed after Masson’s trichrome staining. The scare size was measured by calculating the ratio of blue (fibrotic) to total myocardial area from at least 20 fields of each heart using Image J.

### Mitochondrial staining

The cells were plated onto the confocal dishes and stained for 20 min with 0.02 µM MitoTracker Red CMXRos (Molecular Probes, USA). Nuclei were stained with DAPI. BID protein expression was stained with primary antibodies against BID (sc-6538). The confocal dishes were imaged with an Olympus confocal microscope. The percentage of cells with mitochondria fission relative to the total number of cells is measured.

### Transmission electron microscopy (TEM)

Heart ultrastructural analyses were performed to quantify mitochondrial fission. Samples were examined at a magnification of 15,000 with a FEI Tecnai G^2^ Spirit transmission electron microscope (FEI Co. Ltd, Hillsboro, Oregon, USA). For comparison of mitochondrial fission, TEM micrographs of thin sections were analyzed. We evaluated the morphology of individual mitochondria using Image Pro Plus software (Silver Spring, MD, USA). Approximately 100 mitochondria in a representative area were measured to determine the percentages of mitochondria with various sizes. Mitochondria disintegrated into numerous small round fragments of varying size in heart upon MI and mitochondria smaller than < 0.6 mm^2^ was categorized as fission mitochondria.

### Chromatin immunoprecipitation (ChIP)

The NRVCs were washed with PBS and incubated with 1% formaldehyde for 10 min at room temperature. We quenched the cross-linking with 0.125 M glycine for 5 min and lysed cells for 1 h at 4 °C with 100 μL lysis buffer. We sonicated the cell lysates into chromatin fragments for 4 min 30 s with an average length of 500 to 800 base pairs (bp). The samples were precleared with Protein-A agarose (Roche), and 5 mg specific antibodies (antibody to p53 (SAB1306667, SIGMA) were added and rocked for overnight at 4 °C. Immunoprecipitates were captured with 10% (vol/vol) Protein-A agarose for 4 h and DNA fragments were purified with a QIAquick Spin Kit (Qiagen). We performed PCR with primers that encompassed p53 BS on the rat miR-181a promoter. The oligonucleotides were as follows: forward, 5′- CAGATGTCCATTTTTAGTC-3′; reverse, 5′- GTTTTTGAATCCCAAACT-3′.

### Statistical analysis

Data are expressed as the mean SEM of at least three independent experiments. Statistical analysis was performed with GraphPad Prism version 9.51 (GraphPad Software, Inc.) and the SPSS 18.0 software package (SPSS Inc.). The Mann–Whitney *U*-test was used for nonparametric data. Data groups (two groups) with normal distribution were compared using two-sided unpaired Student’s *t-*test. A one-way ANOVA analysis of variance was used for multiple comparisons. A value of *P* < 0.05 was considered significant.

### Additional methods

The cardiac-specific miR-181a transgenic (Tg) mice generation, in vivo gene transfer of miR-181a antagomir and adenoviruses harboring p53 siRNA, synthesis of miRNAs and sequences of miRNA antagomir, quantitative real-time PCR (qRT-PCR), western blot analysis, reporter constructions and luciferase assay, siRNA constructions of PDCD4, BID, p53, preparations of PDCD4 and p53 overexpression constructs are described in the Supplementary Methods.

## Results

### miR-181a inhibits apoptosis and MI

**Figure 1:**
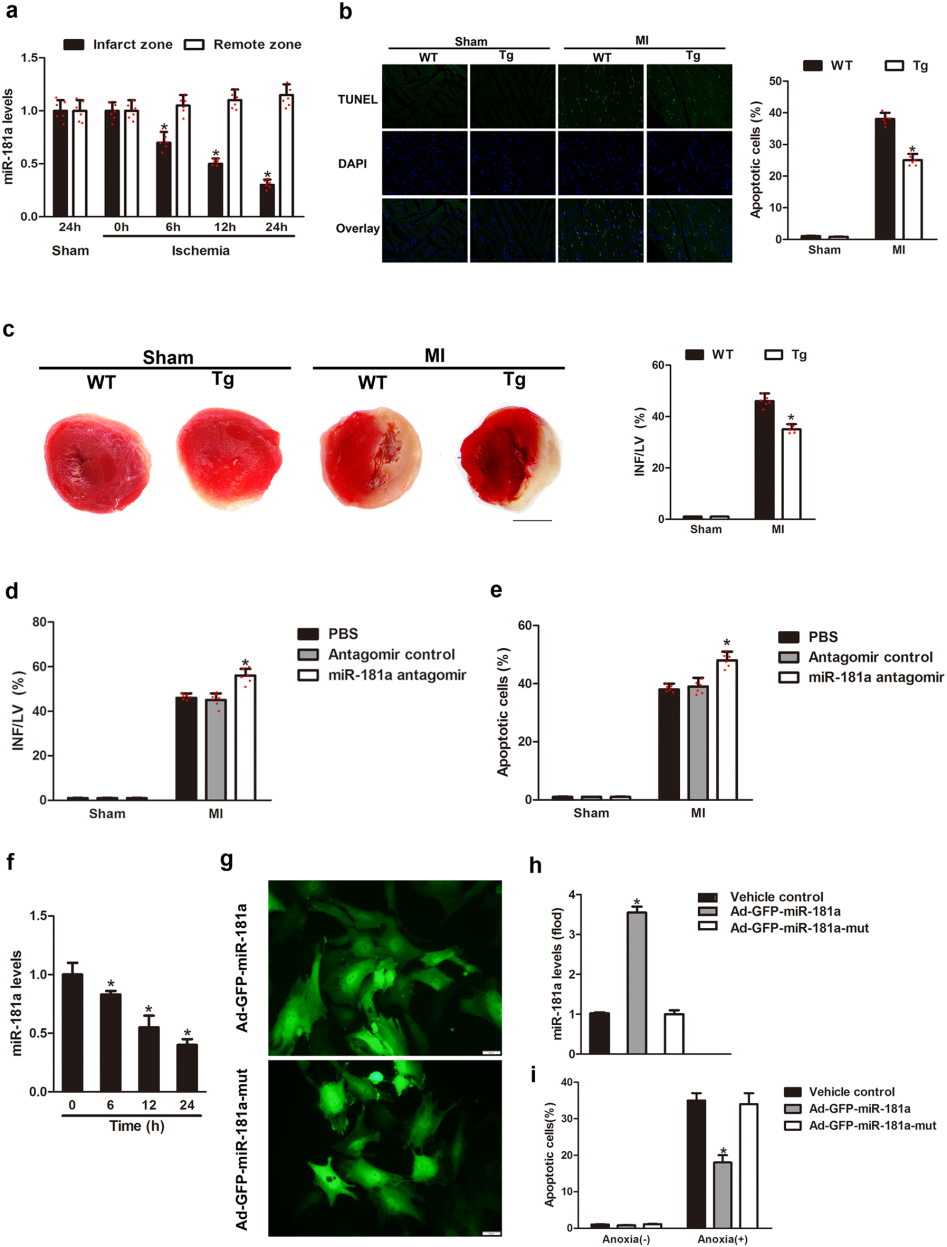
miR-181a inhibits apoptosis and MI. (**a**) miR-181a expression of the mouse heart from ischemic injury after MI. (**b**) Representative images of cardiomyocytes in heart sections from WT and miR-181a Tg mice. Green, TUNEL-positive myocyte nuclei; blue, DAPI-stained nuclei; original magnification, × 400. Quantitative analysis of apoptosis is shown at right. n = 6 mice per group; *P < 0.05 compared with WT. **(c**) Representative images of TTC-stained heart slices and infarct sizes from WT and Tg mice after MI or sham operation. INF, infarct area, LV, left ventricular area. The ratios of INF to LV are shown. *n* = 6 mice per group; *P < 0.05 compared with WT. Scale bar, 2 mm. (**d, e**) Ischemia-induced apoptosis of cardiomyocytes determined by TUNEL (d) and MI (e) treated with PBS, or antagomir control, miR-181a antagomir. *n* = 6 mice per group. *P < 0.05 compared with antagomir control. (**f**) miR-181a expression in NRVCs induced by anoxia. *P < 0.05 compared with control. (**g**,**h**) Representative images of NRVCs (g) and miR-181a levels (h) infected with adenoviral construct encoding green fluorescent protein (GFP) and miR-181a (Ad-GFP-miR-181a) or its mutated form (Ad-GFP-miR-181a-mut). *P < 0.05 compared with Ad-GFP-miR-181a-mut. Scale bar, 50 μm. (**i**) Cell apoptosis of NRVCs infected with Ad-GFP-mir-181a or Ad-GFP-miR-181a-mut and then induced by anoxia. *P < 0.05 compared with Ad-GFP-miR-181a-mut.

We first investigated the potential role of miR-181a in myocardial ischemia injury using miR-181a Tg mice (supplementary Fig. 1). In the infarcted zone of the ischemic heart, miR-181a was found to have lower levels of expression as compared to those in the remote zone (Fig. 1a). When compared to those from wild-type (WT) littermates, miR-181a Tg mice showed a significant decrease in the amount of ischemia-induced apoptosis after 24 h following MI, as measured by TUNEL staining (Fig. 1b) and myocardial infarct size, which decreased from 46.1% to 35.2% of INF/LV area by TTC staining (Fig. 1c). To speculate the effects of endogenous miR-181a on apoptosis, we performed Lipofectamine-mediated in vivo transfection method to reduce miR-181a in the heart of WT mice^17^. Knockdown of miR-181a increased MI size at INF/LV (Fig. 1d). The percentage of cardiomyocytes that had undergone apoptosis was also shown to be higher in animals that had been treated with miR-181a antagomir rather than mice that had been treated with antagomir as a control (Fig. 1e).

To test miR-181a levels under pathological conditions, we found that the expression of miR-181a was decreased in NRVCs under anoxia conditions (Fig. 1f). NRVCs were transduced with either Ad-GFP-miR-181a encoding miR-181a or Ad-GFP-miR-181a-mut encoding a miR-181a mutant as control (Fig. 1g,h). Ad-GFP-MiR-181a, but not Ad-GFP-miR-181a-mut antagonized NRVCs apoptosis induced by anoxia (Fig. 1i). According to these findings, miR-181a is responsible for preventing myocardial ischemia-induced apoptosis as well as MI.

### miR-181a overexpression protects LV function after MI

**Figure 2:**
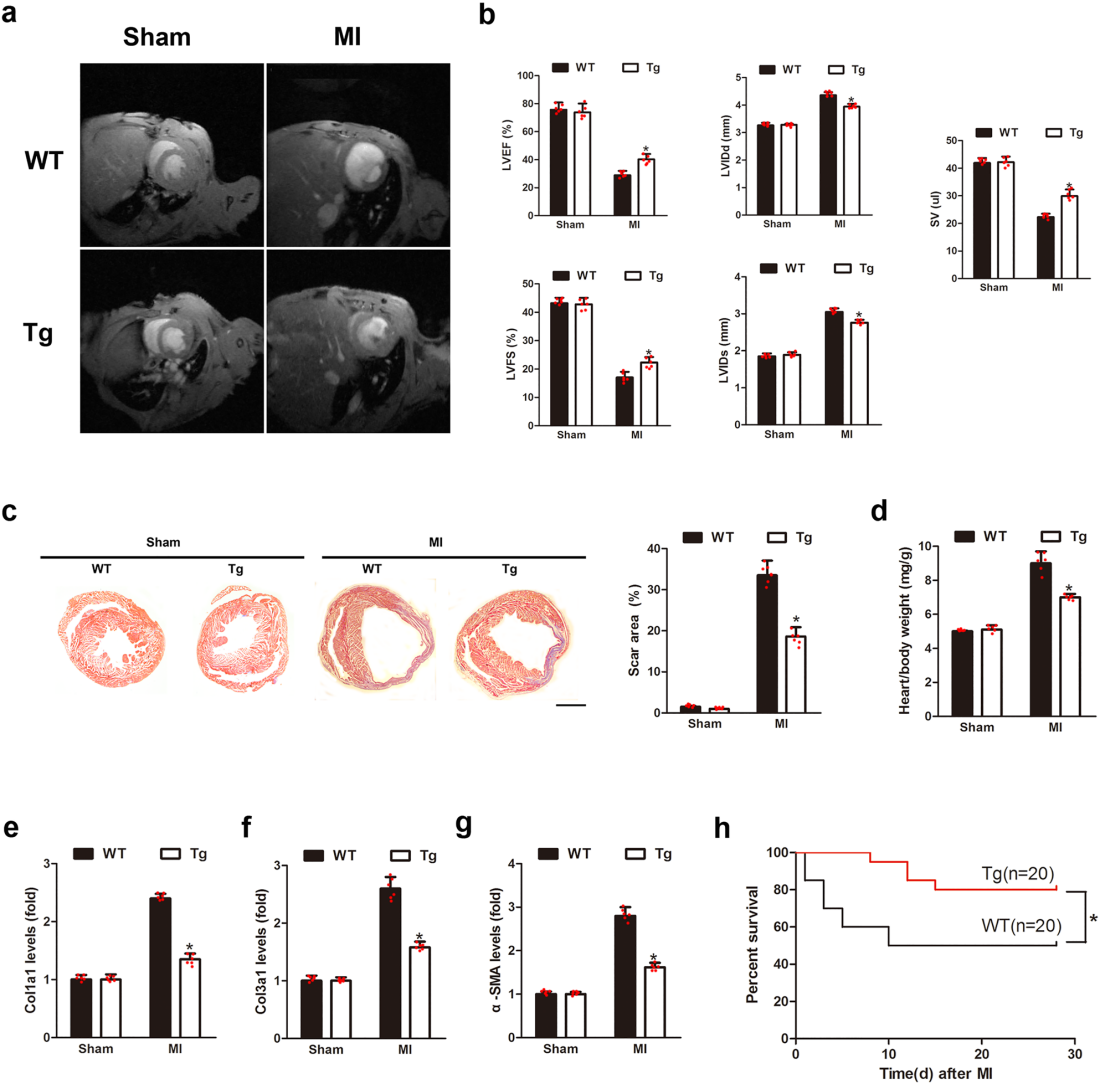
miR-181a overexpression protects mouse hearts from MI-induced LV remodeling. (**a**) Representative end-systolic short-axis cine MR images of WT and miR-181a Tg mice at 4 weeks after MI, respectively. (**b**) MRI analysis **LV** dimensions and cardiac function in WT and miR-181a Tg mice at 4 w after sham or MI. LVEF, left ventricular ejection fraction; LVFS, left ventricular fractional shortening; LVIDd, diastolic left ventricular internal diameters; LVIDs, systolic left ventricular internal diameters; SV, systolic volume. *P < 0.05 compared with WT subjected to MI; n = 6 mice per group. (**c**) Representative photographs and quantitative data of Masson’s trichrome staining of heart sections for collagen 4 weeks after MI with WT and miR-181a Tg mice. *P < 0.05 compared with WT subjected to MI; Scale Bar, 1.5 mm. n = 6 mice per group. (**d**) Heart/body weight ratio of WT and miR-181a Tg mice subjected to 4 weeks MI or sham. *P < 0.05 compared with WT subjected to MI; n = 6 mice per group. (**e**) Kaplan–Meier survival curves of WT and miR-181a Tg mice subjected to 4 weeks MI. *P < 0.05 compared with WT subjected to MI; n = 20 mice per group. (**f**) qRT-PCR showing mRNA levels of col1a1, col3a1, α-SMA in hearts of WT or miR-181a Tg mice subjected to 4 weeks MI or sham. *P < 0.05 compared with WT subjected to MI; n = 6 mice per group.

Acute myocardial infarction (AMI) is a severe cardiovascular event that causes in remodeling of the left ventricle (LV) and malfunction of the heart^18^. For the purpose of determining whether or not miR-181a cardiac overexpression can attenuate LV remodeling and improve cardiac function in the post-MI heart, we used cardiac MRI to analyze LV size and global function serially after 4 weeks of MI in both control and cardiac-specific miR-181a Tg mice. MRI revealed that miR-181a Tg mice preserved cardiac size and function as assessed by an increase in LVEF, LVFS, SV and LVIDs, LVIDd (Fig. 2a,b) than control mice after 4 weeks post-MI. Consistent with the reduction of cardiac dysfunction, the overexpression of cardiac-specific miR-181a resulted in a smaller scar size and hypertrophy 4 weeks after MI to prevents cardiac remodeling (Fig. 2c,d). miR-181a Tg mice also reduced the gene levels on fibrosis compared with control mice (Fig. 2e–g). There was higher chronic MI-induced survival in miR-181a Tg mice than WT littermate controls (Fig. 2h). Knockdown of endogenous miR-181a by carboxyfluorescein (FAM)-labeled antagomir displayed significantly impaired cardiac function and structure following MI characterized by heart MRI parameters and collagen content (Supplementary Fig. 2, 3, 4). As a result, increased expression of miR-181a in the mouse heart is linked to enhanced LV function and remodeling.

### *PDCD4 is the miR-181a target gene that is involved in miR-181a-mediated anti*-*ischemic injury*

**Figure 3:**
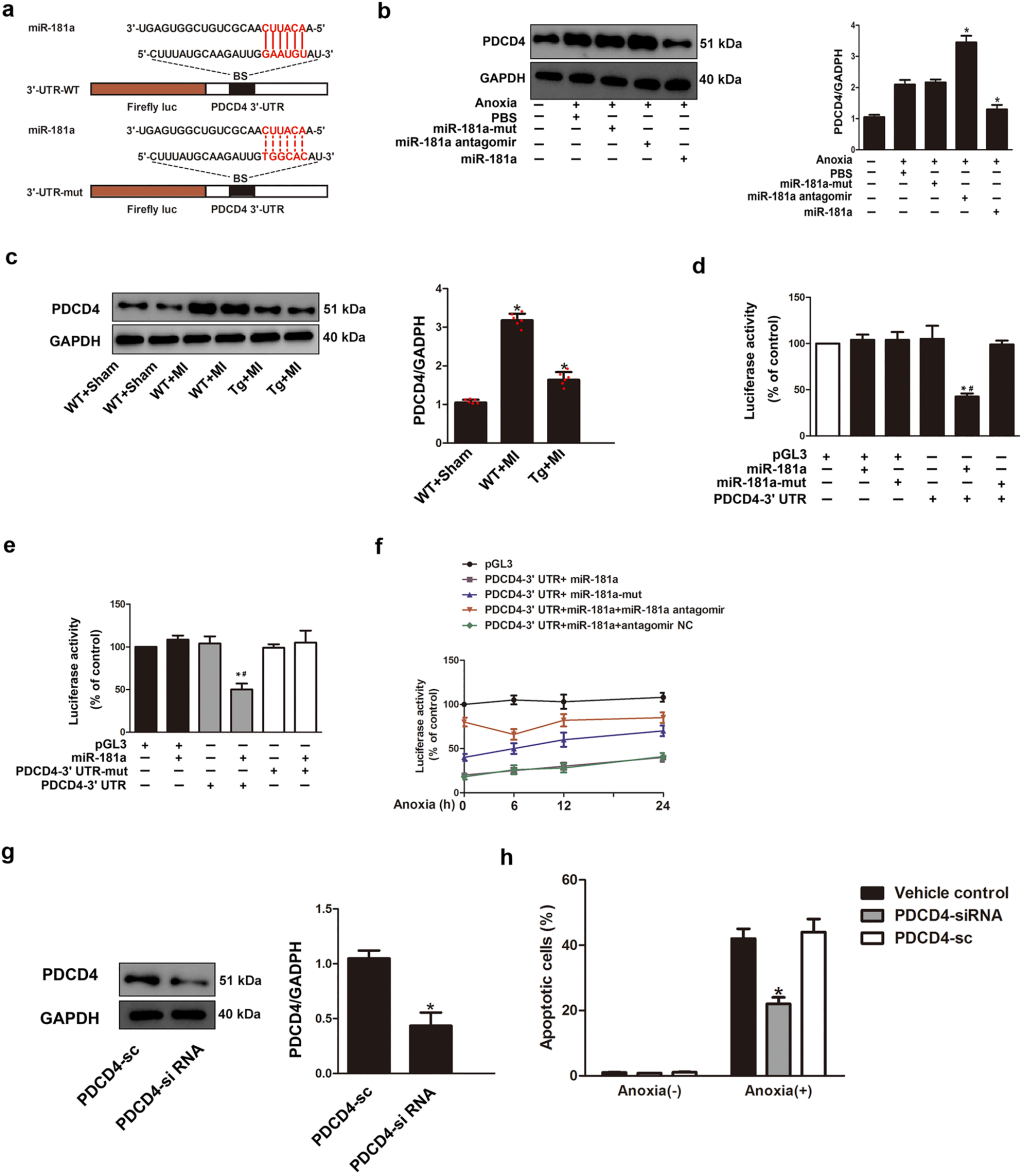
PDCD4 is a miR-181a target gene that is involved in miR-181a-mediated anti-ischemic injury. (**a**) Putative miR-181a binding sites in the 3’-UTR of *PDCD4* mRNA. Potential complementary residues are highlighted in red, and base pairing between miR-181a and target site marked by vertical lines. (**b**) Expression of PDCD4 protein was detected using western blot assay in NRVCs exposed to anoxia transfected with miR-181a or the miR-181a antagomir (left) and quantification of PDCD4 normalization to GAPDH (right). *P < 0.05 compared with anoxia alone. Results are representative of three independent experiments. (**c**) miR-181a Tg mice exhibit a low level of PDCD4 upon MI (n = 6). The expression of PDCD4 protein was detected by western blot. *P < 0.05 compared with WT mice upon MI. Results are representative of three independent experiments. (**d**) Ability of miR-181a to directly repress activity of the luciferase reporter construct that contains the portion of the PDCD4 3’-UTR. *P < 0.05 compared with transfection of PDCD4-3’UTR construct alone. ^#^*P* < 0.05 compared with transfection of miR-181a-mut with PDCD4-3’ UTR construct alone. (**e**) Introduction of mutations in the 3’UTRs of PDCD4 blocks the inhibitory effect of miR-181a on their translation activity. *P < 0.05 compared with transfection of PDCD4 3’UTR construct alone. ^#^*P* < 0.05 compared with transfection of miR-181a with PDCD4-3’UTR-mut construct. (**f**) Anoxia promotes the translation activity of PDCD4-3’UTR inhibited by miR-181a. Luciferase activity measured from NRVCs infected with adenoviral miR-181a or miR-181a-mut, and transfected with a luciferase construct containing the PDCD4-3’UTR, the miR-181a antagomir or miR-181a negative control (NC), and exposed to anoxia. (**g**) Expression of PDCD4 protein was detected using western blot and quantification of PDCD4 normalization to GAPDH. *P < 0.05 compared with PDCD4-sc. (**h**) The percentage of cardiomyocytes apoptosis after knockdown of PDCD4 with siRNA then induced by anoxia. *P < 0.05 compared with PDCD4-sc.

MiRDB, PicTar, and Targetscan, were used to examine the probable targets of miR-181a, which regulates apoptosis and protects against ischemia injury. We focused on PDCD4, an apoptosis-related gene that was identified as a potential target of miR-181a by all three target prediction algorithms. PDCD4 has been suggested to underlie cardiomyocyte apoptosis induced by myocardial ischemia injury and progressive ventricular remodeling^19^. The binding sites of miR-181a were well conserved among PDCD4 mRNAs from mouse, rat and human (Fig. 3a). We then confirmed that miR-181a-mediated anti-ischemic damage targets PDCD4. Western blot result showed that adenoviral miR-181a reduced PDCD4 protein expression, while miR-181a antagomir therapy increased PDCD4 protein levels (Fig. 3b). After 24 h post-MI, there was a considerable increase in the levels of protein expression of PDCD4 in the hearts of mice, whereas cardiac-specific miR-181a Tg mice decreased PDCD4 protein expression levels in heart at 24 h post MI compared with WT littermate controls (Fig. 3c). Data from the luciferase reporter assay showed that overexpression of miR-181a, but not miR-181a-mut, significantly suppressed luciferase activity (Fig. 3d). Furthermore, miR-181a decreased the translation level of the wild type PDCD4 3’-UTR, while the PDCD3’-UTR-mut did not respond significantly to miR-181a (Fig. 3e). Overexpression of miR-181a suppressed the anoxia-induced rise in the activity of PDCD4 3’-UTR luciferase constructs (Fig. 3f). This evidence suggests that PDCD4 is a direct target of miR-181a. Next, we checked the hypothesis that PDCD4 as a target of miR-181a might regulate the apoptotic pathway that is triggered when cells are exposed to anoxia. We discovered that lowering the amounts of PDCD4 protein with small interfering RNA (siRNA) reduced the amount of apoptosis that cardiomyocytes experienced in response to anoxia (Fig. 3g,h). Thus, our data indicate that downregulation of pro-apoptotic PDCD4 is partly involved in miR-181a-mediated anti-ischemic injury in the heart.

### PDCD4 and BID participate in the regulation of apoptosis and mitochondrial fission in cardiomyocytes

**Figure 4:**
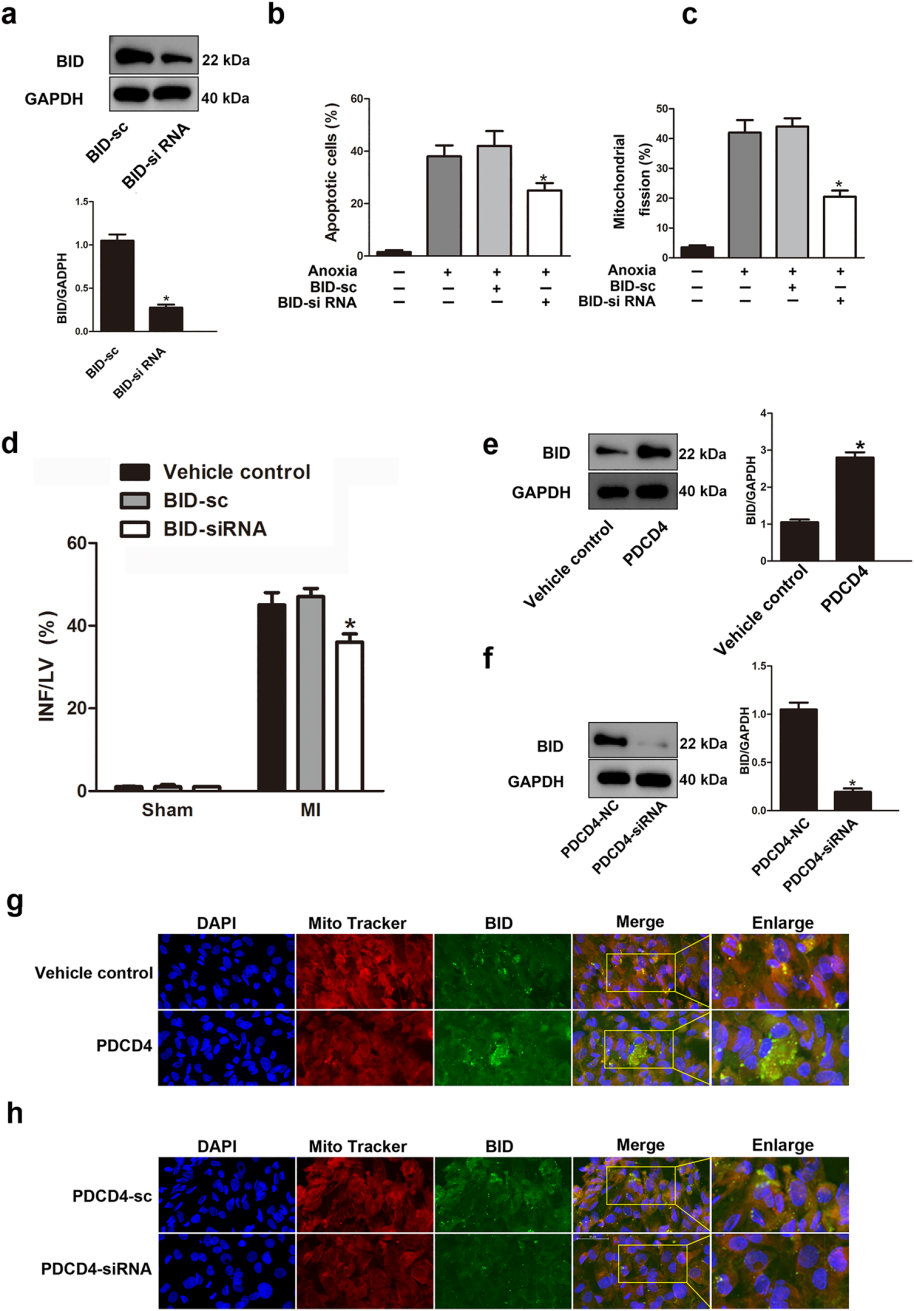
PDCD4 and BID participate in the regulation of apoptosis and mitochondrial fission in cardiomyocytes. (**a**) Expression of BID protein was detected using western blot. *P < 0.05. (**b**) Cell death in cardiomyocytes transfected as in (**a**) and then exposed to anoxia. *P < 0.05 compared with anoxia alone. (**c**) Mitochondrial fission in NRVCs transfected as in (a) and then exposed to anoxia. *P < 0.05 compared with anoxia alone. (**d**) INF/LV in hearts of mice infected with BID-siRNA or BID-sc at 24 h induced by myocardial infarct. n = 6 mice per group; *P < 0.05 compared with BID-sc. (**e**,**f**) Expression of BID protein was detected using western blot assay. *P < 0.05. (**g**,**h**) Representative images of subcellular localization of BID in NRVCs. Scale bar, 50 μm.

Furthermore, we evaluated whether PDCD4 and BID involving mitochondrial fission engage in the regulation of apoptosis in cardiomyocytes post MI to learn more about the pathophysiological significance of PDCD4 in myocardial ischemia injury. Silencing of genes using siRNAs led to a dramatic decrease in BID protein production (Fig. 4a). Knockdown of BID inhibited cardiomyocytes apoptosis and mitochondrial fission treated with anoxia in vitro (Fig. 4b,c). Knockdown of BID attenuated INF/LV in hearts of mice in vivo (Fig. 4d). To determine that the subcellular distribution and recruitment of BID mediated by PDCD4 in mitochondrial, following the transfection of NRVCs with PDCD4-overexpression constructs or PDCD4-siRNA, the cells were subsequently subjected to anoxia. Western blot analysis showed that PDCD4-overexpression plasmid enhanced BID protein expression, but PDCD4-siRNA decreased it (Fig. 4e,f). Overexpression of PDCD4 in NRVCs upon anoxia increased BID recruitment to the mitochondria, while silencing by siRNA prevented it (Fig. 4g,h). Anoxia appears to translocate BID to NRVC mitochondria. PDCD4 knockdown prevented anoxia-induced mitochondrial fission and apoptosis (Supplementary Fig. 6a). BID knockdown reduced PDCD4 overexpression-induced mitochondrial fission and apoptosis (Supplementary Fig. 6b,c). These results support the hypothesis that anoxia-induced mitochondrial fission and death in cardiomyocytes are controlled by PDCD4-mediated BID.

### miR-181a regulates apoptosis and mitochondrial fission through PDCD4 and BID

**Figure 5:**
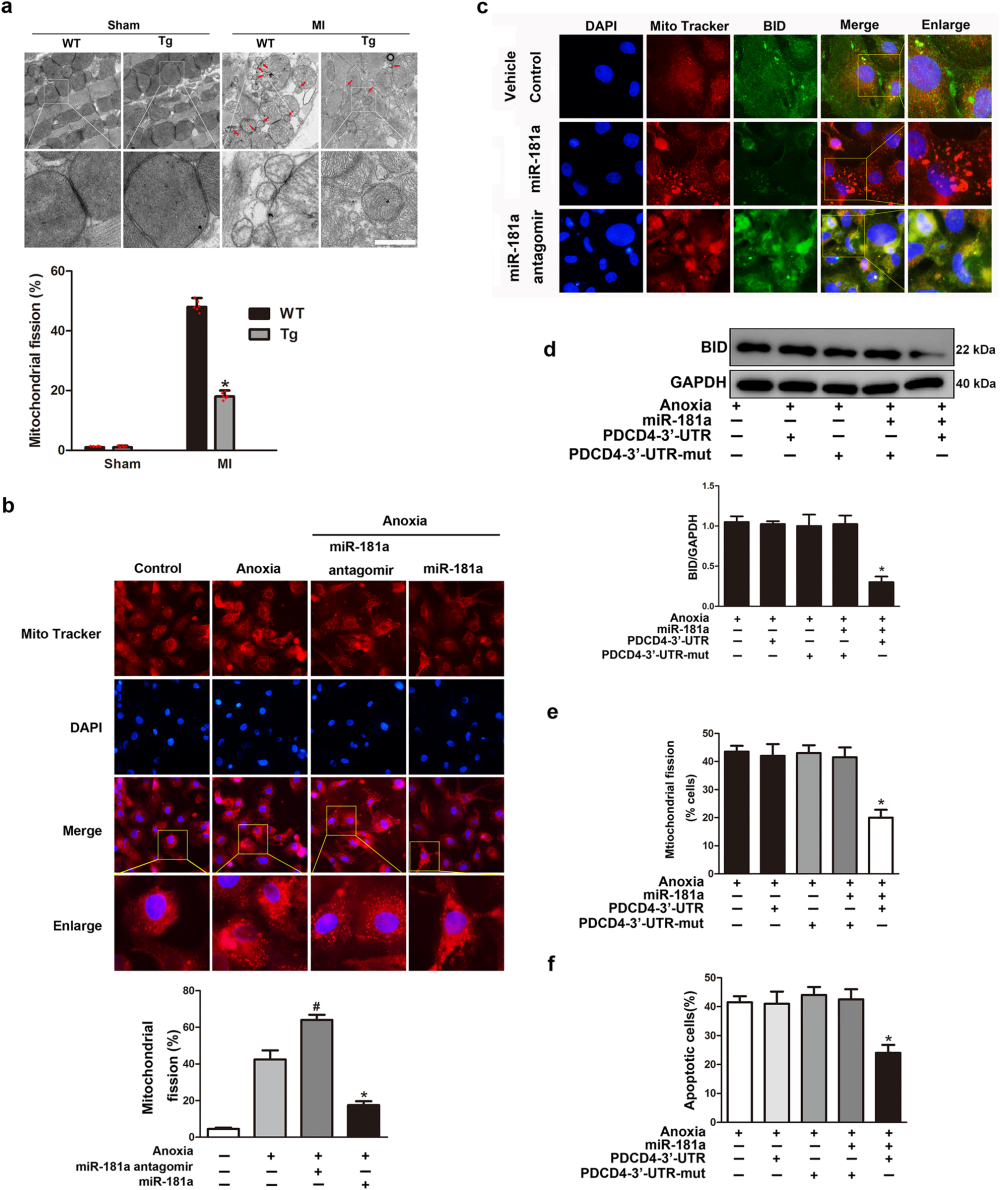
miR-181a regulates apoptosis and mitochondrial fission through PDCD4 and BID. (**a**) Representative TEM images of mitochondria from miR-181a Tg mice or WT mice subjected to MI, Scale bar, 0.5 μm; arrows indicate fission mitochondria. n = 6 mice per group. (**b**) Representative mitochondrial morphology images in cardiomyocytes infected with adenoviral miR-181a or miR-181a antagomir (up). Original magnification, × 400. Quantitative analysis of mitochondrial fission is shown at down. *P < 0.05 compared with anoxia alone. (**c**) Representative images of subcellular localization of BID in NRVCs transfected with adenoviral miR-181a or miR-181a antagomir and then exposed to anoxia. Original magnification, × 400. (**d**) Expression of BID protein was detected using western blot assay. *P < 0.05 compared with adenoviral miR-181a along with PDCD4-3’UTR-mut. (**e**) Mitochondrial fission in NRVCs upon to anoxia co-transfected with adenoviral miR-181a in combination with PDCD4-3′UTR or PDCD4-3′UTR-mut. *P < 0.05 compared with adenoviral miR-181a along with PDCD4-3′UTR-mut. (**f**) Apoptotic cells in NRVCs upon to anoxia co-transfected with adenoviral miR-181a in combination with PDCD4-3′UTR or PDCD4-3′ UTR-mut. *P < 0.05 compared with adenoviral miR-181a along with PDCD4-3’UTR-mut.

We initially analyzed mitochondrial fission in vivo upon MI to determine whether miR-181a controls anoxia-induced apoptosis and mitochondrial fission via PDCD4 and BID. By using TEM, we found no difference in the mitochondrial morphology of the hearts of sham-operated WT and miR-181a Tg mice. MI led to an increase in mitochondrial fission in both WT and miR-181a Tg mice, whereas this effect was attenuated in miR-181a Tg mice (Fig. 5a). Furthermore, miR-181a overexpression prevented mitochondria fission in NRVCs exposed to anoxia, but miR-181a antagomir significantly exacerbated mitochondrial fission in vitro (Fig. 5b). Next, we tested whether miR-181a influences BID translocation to mitochondria through its effects on PDCD4 expression. Overexpression of miR-181a suppressed BID expression and prevented BID translocation to mitochondria; however, the miR-181a antagomir increased BID expression and restored BID translocation to mitochondria (Fig. 5c). Co-transfection with WT PDCD4-3’ UTR, but not PDCD4-3’ UTR-mut, resulted in a decrease in BID protein expression after miR-181a overexpression, as shown by western blotting assay (Fig. 5d). When WT PDCD4-3’ UTR was present, overexpression of miR-181a slowed down mitochondrial fission and cell death. This did not happen when PDCD4-3′ UTR was changed (Fig. 5e,f). Further, overexpression of miR-181a alone did not influence the mitochondrial fission (Supplementary Fig. 7). Together, these data demonstrate that miR-181a modulates anoxia-induced apoptosis in cardiocytes via targeting BID and PDCD4.

### p53 regulates miR-181a expression

**Figure 6:**
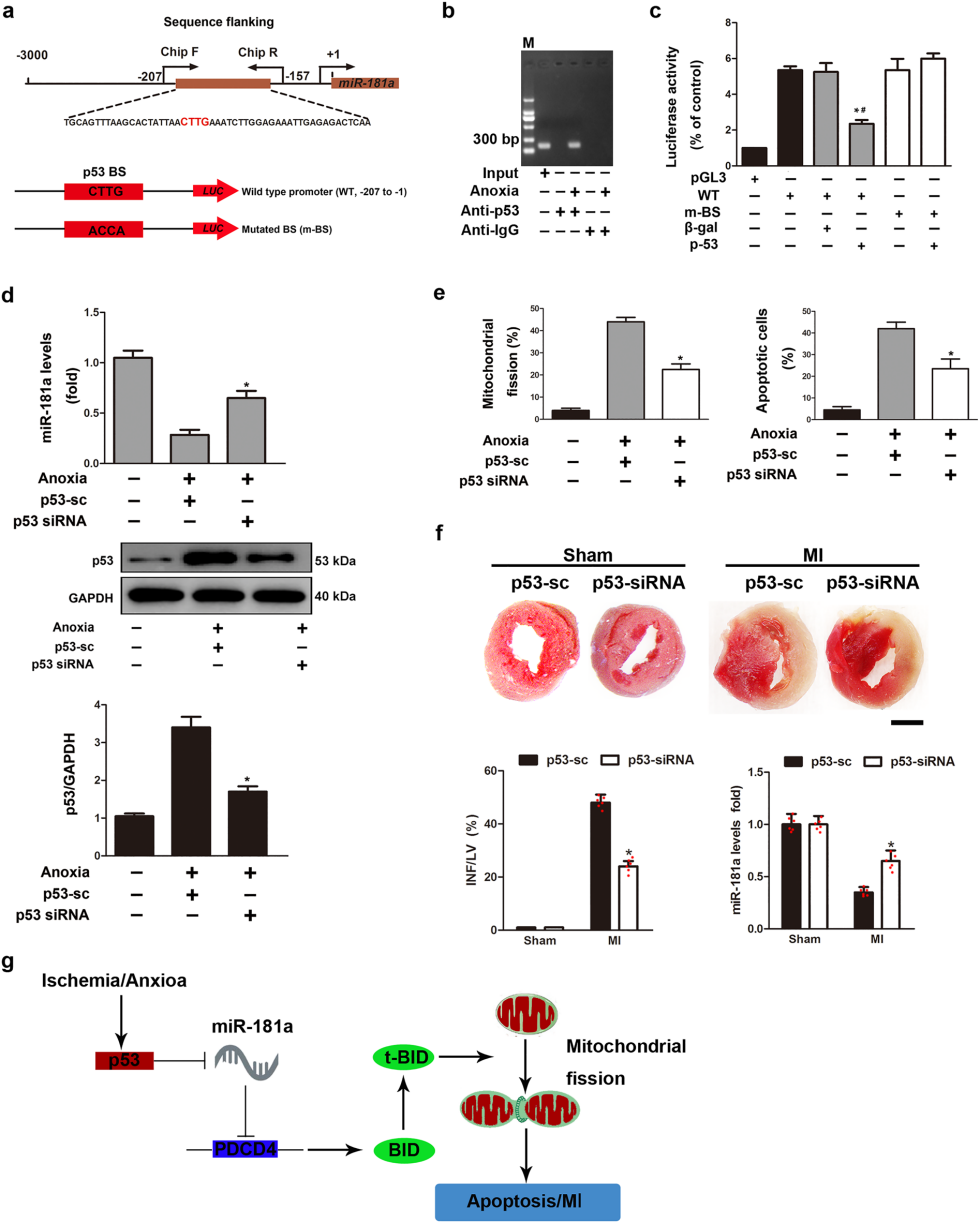
p53 transcriptionally downregulates miR-181a upon to anoxia. (**a**) Schematic representation of rat miR-181a promoter region containing a potential p53-binding site. (**b**) ChIP analysis of p53 binding to the miR-181a promoter in NRVCs treated or not with anoxia. The anti-IgG antibody was used as a negative control. (**c**) Luciferase activity measured from NRVCs infected with adenovirus harboring p53 or β-galactosidase (β-gal) and transfected with empty vector (pGL3) or with constructs containing the WT miR-181a promoter or the miR-181a promoter mutated the putative p53 binding sites (m-BS). Firefly luciferase activities were normalized to Renilla luciferase activities. *P < 0.05 compared with WT construct alone; ^#^*P* < 0.05 compared with m-BS construct in combination with p53. (**d**) miR-181a levels by RT-PCR and p53 determined by in NRVCs transfected with adenoviral p53 siRNA or its scrambled form (p53-sc) as control. *P < 0.05 compared with control. (**e**) NRVCs upon anoxia or not were infected with adenoviral p53 siRNA or its control p53-sc and the percentage of cells with mitochondrial fission or cell apoptotic was determined. *P < 0.05 compared with control. (**f**) miR-181a levels by RT-PCR and myocardial infarct sizes in the hearts of mouse subjected to MI or not and infected with adenoviral p53 siRNA or its control p53-sc. n = 6 mice per group. Left graph, *P < 0.05 compared with control. Right graph, *P < 0.05 compared with control subjected to MI. Scale bar, 2 mm. (**g**) Schematic model of miR-181a’s role in regulating mitochondrial fission and cardiomyocytes apoptosis.

How might MI downregulate miR-181a expression? P53, which can repress gene expression and contribute to MI, may influence the expression of miRNA at the transcriptional level under pathological circumstances. We analyzed the promoter region of rat miR-181a and found a possible binding site (BS) of p53, which led us to hypothesize that p53 plays a role in the control of miR-181a expression during MI (Fig. 6a). ChIP research showed that, in the presence of oxygen deprivation, p53 linked to the BS of the miR-181a promoter. We found that miR-181a promoter was greatly enriched by the p53 antibody with anoxia treatment (Fig. 6b). These results show that p53 can bind to the miR-181a promoter, but that hypoxia therapy reduces this binding.

The ability of p53 to regulate miR-181a promoter activity was then investigated. The promoter activity of WT miR-181a was shown to be inhibited by p53 using a luciferase assay. Nevertheless, mutations put into the BS region abolished this inhibition (Fig. 6c). Moreover, knockdown of p53 by siRNA abolished the reduction of miR-181a levels in NRVCs upon anoxia (Fig. 6d). The BS of the miR-181a promoter is in the intron of the Nr6a1 gene, but *Nr6a1* mRNA levels were not significantly changed by ischemic injury (Supplementary Fig. 8a), anoxia (Supplementary Fig. 8b), or p53 overexpression (Supplementary Fig. 8c), or in miR-181a Tg mice (Supplementary Fig. 8d). These results show that p53 can halt the transcription of miR-181a.

Anoxia-induced apoptosis and mitochondrial fission in cardiomyocytes were decreased by p53 knockdown (Fig. 6e), while BID knockdown blunted p53's effects on mitochondrial fission and apoptosis (Supplementary Fig. 9). The knockdown of p53 in the hearts of mice that were given a MI attenuated the reduction in the levels of miR-181a, and also reduced the extent of the infarct in vitro (Fig. 6f). Additionally, anoxia increased the activity of a luciferase reporter construct harbouring the PDCD4-3’ UTR, and knockdown of p53 suppressed this effect (Supplementary Fig. 10). Our results imply that miR-181a is transcriptionally downregulated by p53 in response to anoxia.

## Discussion

Mitochondria are abundant in cardiomyocytes and mitochondrial dynamic is an important determinant of organelle fidelity and cardiac homeostasis. However, alterations in mitochondrial fission and fusion may influence cellular sensitivity to apoptotic cell death, a factor in cardiac ischemic/anoxic injury-induced cell death^20^. MI causes dysfunction in energy metabolism, oxidative stress, and apoptosis, all of which are regulated in part by abnormal mitochondrial fission and fission^21^. By modulating processes as diverse as apoptosis, respiration, mitosis, and development, inhibiting mitochondrial fission protects the heart from ischemia injury^22^. Ischemic heart disease has long been known to have the machinery for controlling mitochondrial fission and fusion, but its function has only recently been understood^22^.

Recent research on miRNAs has provided an up-to-date understanding of how mitochondrial fission is involved in controlling apoptosis in cardiomyocytes. Targeting calcineurin and dynamin-related protein-1, miR-499 controlled mitochondrial dynamics and protected the heart from MI^5^. Mitochondrial fission was mediated by miR-30 via p53 and the dynamin-related protein-1 pathway^23^. In cardiomyocytes, miR-484 inhibits Fis1-mediated fission and death by suppressing translation of the mitochondrial fission protein Fis1^24^. miR-361 prevents mitochondrial fission and apoptosis, and thereby shields the heart from ischemia injury^25^. Our results show that miR-181a has a role in preventing anoxia- and ischemia-induced mitochondrial fission, cardiomyocyte apoptosis, and MI. To explore further about miR-181a, we searched for the proteins it interacts with and found that PDCD4, a critical anti-apoptotic factor, is one of its downstream targets. The effects of miR-21, miR-24, miR-210, and miR-499 on anti-apoptotic and anti-ischemia injury are comparable to those seen in the present investigation^5,26–28^.

Identifying miRNAs that affect mitochondrial dynamics and apoptosis in the heart and characterizing their signal transduction routes in the apoptotic cascades are of utmost importance. Here, our results demonstrates that myocardial ischemia induces p53 transcriptionally, which may govern the miR-181a-modulated mitochondrial fission and apoptotic pathway involving PDCD4 and BID. PDCD4 is elevated in anoxia and ischemia, consistent with earlier research indicating PDCD4 rises under pathological situations such MI and ischemic reperfusion injury and that inhibiting PDCD4 can reduce MI^10^. A recent investigation has shown that PDCD4 actively affects Bid expression, which is a key factor in the regulation of apoptosis in H_2_O_2_-exposed cardiomyocytes^29^. PDCD4 enhanced BID recruitment to mitochondria and promoted mitochondria fission and apoptosis of the cardiomyocytes induced by H_2_O_2_^29^. MiR-181a's modulation of the PDCD4-BID apoptotic pathway during ischemia and anoxic conditions may be significant in reducing the effect of pathological damage to the heart after MI.

Bid, a member of the Bcl-2 family that promotes cell death when activated by apoptosis stimuli including ischemia and anoxia, plays a vital role in tissue homeostasis and apoptosis^30^. The beginning of mitochondrial fission and ensuing apoptosis is caused by the interaction of proapoptotic BID with the mitochondrial membrane^31^. Translocation of Bid to mitochondria triggers a cascade of events that culminates in cell death. These processes include the synthesis of mitochondrially active Bid, the release of cytochrome c, and the activation of caspase-3^32^. Our results revealed that Knockdown of BID inhibited cardiomyocytes death and mitochondrial fission treated with anoxia in vitro and attenuated INF/LV in hearts of mice in vivo*.*

Many human disorders, such as cancer, pulmonary hypertension, and ischemic heart disease, share PDCD4 as a critical regulatory target and proapoptotic factor^33,34^. Future antiapoptotic cancer therapeutics may focus on the PDCD4 protein due to its ability to suppress neoplastic transformation^35^. When activated, the PDCD4/caspase-3 axis in pulmonary tissues promotes endothelial apoptosis and pulmonary hypertension^33^. Cellular injury response modelling generated by H_2_O_2_ revealed the role of PDCD4 as a mediator in the anti-apoptotic action of miR-21 on vascular smooth muscle cells^36^. PDCD4 is further verified as a miR-21 target gene, inhibits cardiac cell apoptosis during acute MI, ischaemia/reperfusion injury, and H_2_O_2_-induced injury^19,26,34^. Based on the results of the current investigation, it is clear that an increase in BID recruitment to mitochondria caused by anoxia occurs when PDCD4 is overexpressed in cardiomyocytes.

Evidence is mounting suggesting p53 plays a role in a wide variety of biological processes, such as DNA replication, DNA damage checkpoints, DNA repair, mitosis, cell death, cardiac dysfunction, and heart failure^37^. However, the role of p53 in cardiomyocytes in the modulation of the apoptotic signaling cascade by miRNAs is still unclear. In this study, we demonstrated that an essential role of p53 is the regulation of miR-181a levels and mitochondrial dynamics, both of which are involved in the process of apoptosis. Our research indicated that p53 knockdown reduced the drop of miR-181a levels, hence reducing mitochondrial fission and apoptosis in NRVCs in response to anoxia in vivo. The knockdown of p53 in the hearts of mice with MI mitigated the decrease in miR-181a levels and reduced in vitro infarct size. Through modulating miR-181a expression, p53 involved in the mitochondrial fission and cardiomyocytes apoptosis pathway.

The transcription patterns of all of the miRNA host genes have been curated from a number of sources to illustrate the geographical, temporal, and physiological regulation of miRNA expression^38^. Insight into the evolution of miRNA transcription is provided by the study of miRNA promoter and enhancer regulatory elements, and miRNA transcriptional networks can be identified quickly^39^. miRNAs expressed from introns of coding genes are independently regulated by transcription factors^40^. MI and ischemia/reperfusion-induced cardiac dysfunction were both accompanied by a decrease in miR-499 expression, which was transcriptionally repressed by p53^5^. Transcription factors E2F1 switches on the expression of miR-421, which leads to more mitochondrial fragmentation, apoptosis, and cardiovascular disease^38^. Consistent with our findings that p53 regulates the expression of miR-181a but not the *Nr6a1* gene, we give novel evidence proving that miR-181a is an intronic miRNA and we identified the binding site of p53 to be within the intron of the *Nr6a1* gene containing miR-181a.

## Conclusions

The findings of the present investigation show that p53, miR-181a, PDCD4, and BID are all connected in some way to the apoptotic programme of the heart after MI (Fig. 6g). To shed light on the part that other p53 targets play in the chain reactions that make up the mitochondrial network and the apoptotic pathways would be a fascinating endeavour. The results of our research will throw new light on the possibility that miR-181a could one day be used as a therapeutic method to treat MI and heart failure.

### Supplementary Information

Supplementary Information 1.

Supplementary Information 2.

Supplementary Information 3.

## Data Availability

All data generated or analyzed during this study are included in this published article and Supplementary Material.

## Abbreviations

ChIP: Chromatin immunoprecipitation
ECG: Electrocardiography
LAD: Left anterior descending coronary artery
LV: Left ventricular
LVEF: LV ejection fraction
LVFS: LV fractional shortening
LVIDd: Diastolic LV internal diameters
LVIDs: Systolic LV internal diameters
MI: Myocardial infarction
miRNA: MicroRNA
NRVC: Neonatal rat ventricular cardiomyocyte
PDCD4: Programmed cell death protein 4
SV: Systolic volume
TEM: Transmission electron microscopy
TTC: 2, 3, 5-Triphenyl-2H-Tetrazolium Chloride
