# Supplementary material for: MiR-181a protects the heart against myocardial infarction by regulating mitochondrial fission via targeting programmed cell death protein 4
Source: Sci Rep. 2024 Mar 19;14:6638. doi: 10.1038/s41598-024-57206-8 (PMC10951332; doi:10.1038/s41598-024-57206-8)
Supplement: Supplementary file 1 — Supplementary Information 1. [file 41598_2024_57206_MOESM1_ESM.docx]

**Supplementary Materials**

**miR-181a protects the heart against** **myocardial infarction by regulating** **mitochondrial fission via targeting programmed cell death protein 4**

Zhu Jianbing^1, 2, 3, #, *^, Wang Qian^4, #^, Zheng Zeqi^1, 2^, Ma Leilei^3^, Guo Junjie^5^, Shi Hongtao^3^, Ying Ru^1,2^, Gao Beilei^1, 2^, Chen Shanshan^1, 2^, Yu Siyang^1, 2^, Yuan Bin^6^, Peng Xiaoping^1, 2, *^, Ge Junbo^3, *^

**Contents**

**Supplementary Figures 1-10**

**Supplementary Methods**


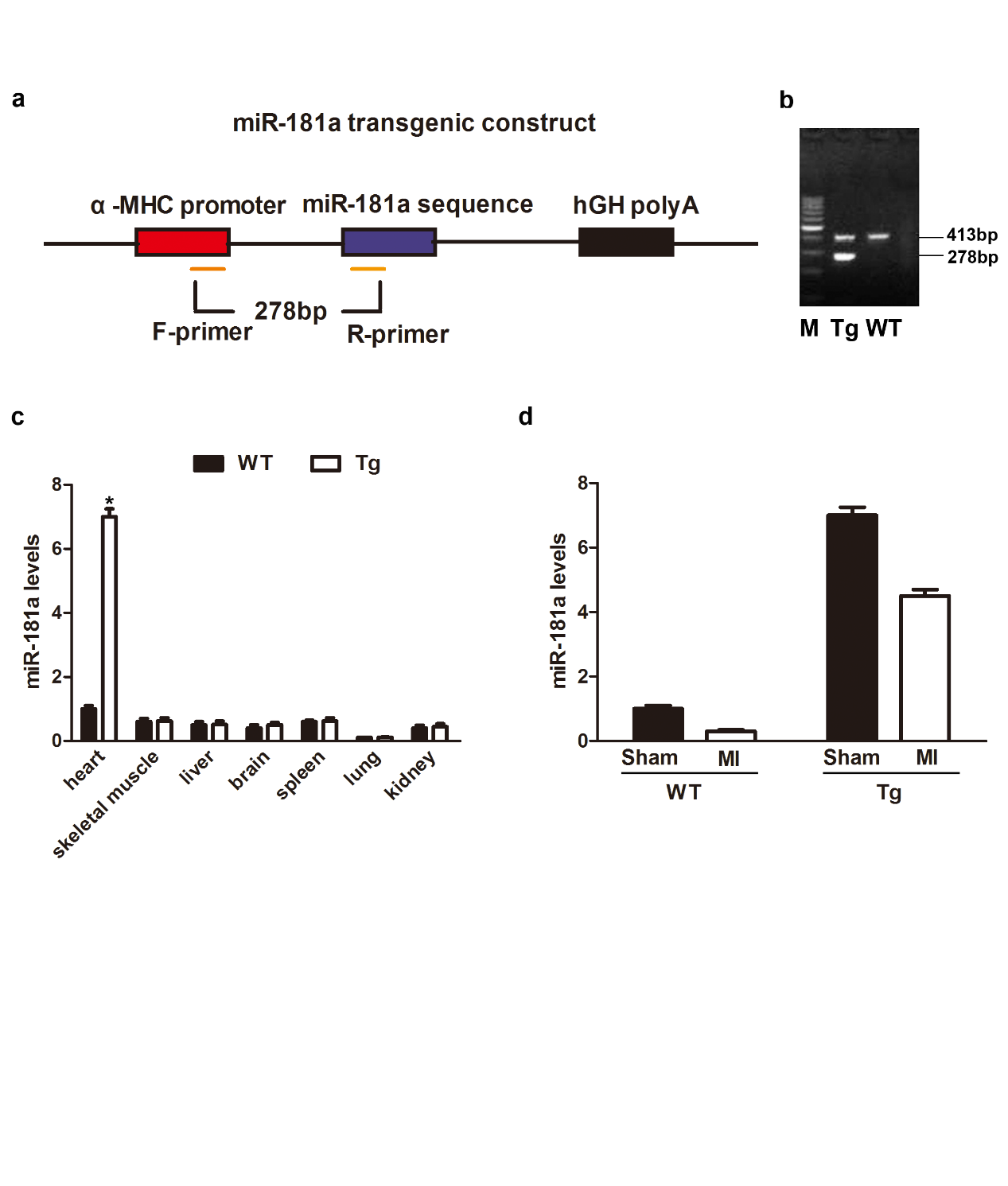


**Supplementary Figure 1. Cardiac-specific miR-181a transgenic mice construct. (a)** Schematic map of the miR-181a transgenic (Tg) mice construct and PCR primers for genotyping Tg mice. **(b)** Genotyping of Tg mice. The positive miR-181a Tg mice but not wild-type (WT) mice express a 278 bp genomic fragment. The internal control PCR targets the endogenous mouse β-actin locus (413 bp). **(c)** qRT-PCR analyzes the levels of miR-181a in different tissues between Tg mice and WT mice. **P* < 0.05 compared with WT mice. **(d)** Tg mice exhibit a high level of miR-181a at 24h after of MI analyzed by qRT-PCR, n = 6.


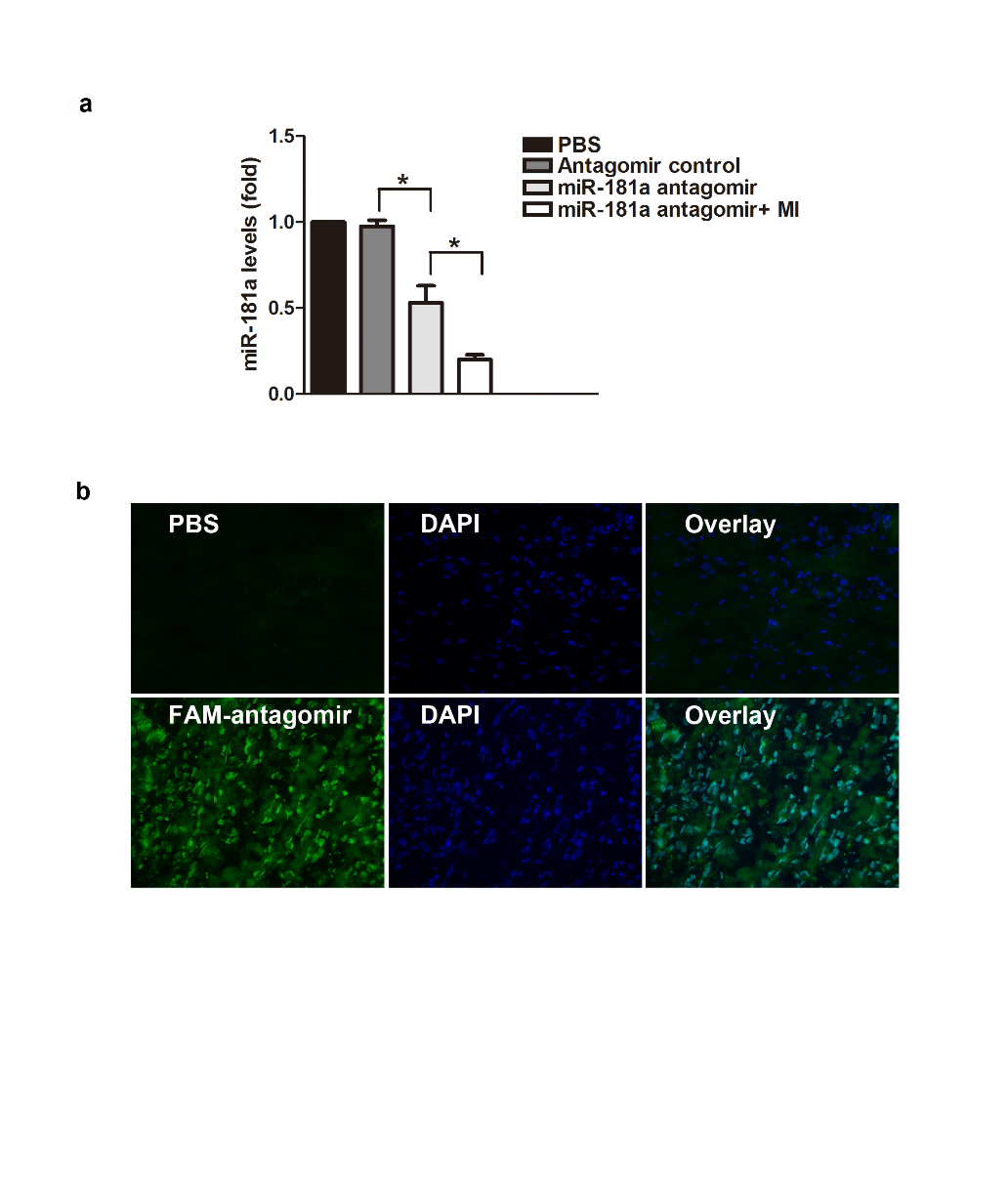


**Supplementary Figure 2. Validation for miR-181a antagomir *in vivo* transfection. (a)** WT mice (8 weeks old) were intramyocardially injected with 80 ng miR-181a antagomir or antagomir control before coronary artery occlusion. 24h after antagomir injection mice were subjected to 24h MI. miR-181a levels were detected by qRT-PCR. **P* < 0.05. n = 6. **(b)** Immunofluorescence showed distributions of carboxyfluorescein (FAM)-labeled antagomirs (Green) in the myocardium at 24h after antagomir injection as described for **(a)**. Cell nuclei were stained with DAPI. Original magnification, ×400.


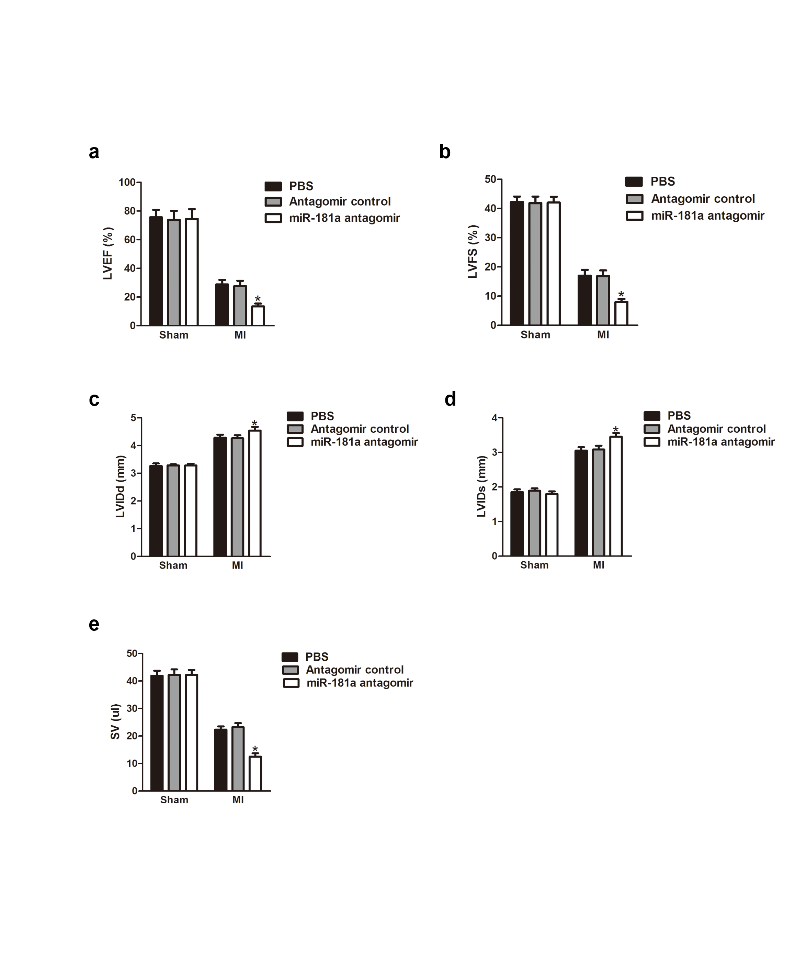


**Supplementary Figure 3. Knockdown of miR-181a aggravates LV dysfunction after myocardial infarction.** MRI analysis left ventricular dimensions and cardiac function in wild-type (WT) and miR-181a transgenic (Tg) mice at 4 weeks after sham or MI. LVEF(**a**), LVFS(**b**), LVIDd (**c**), LVIDs (**d**), SV (**e**). **P* < 0.05 compared with WT subjected to MI, *n* = 6.


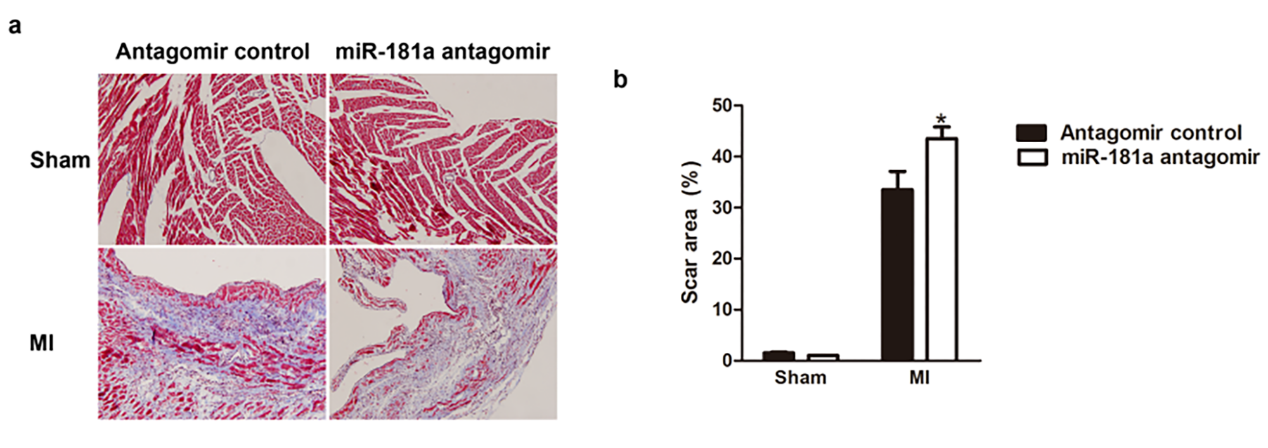


**Supplementary Figure 4. miR-181a antagomir exacerbates the LV remodeling on 4 weeks after MI.** (**a**) Representative Masson’s Trichrome staining images of infarct area on 4 weeks following MI were shown in both miR-181a knockdown and control littermates. (**b**) Quantification of infarct area on 4 weeks following MI in both miR-181a knockdown and control littermates. **P* < 0.05, n = 6 per group. Original magnification, ×200.


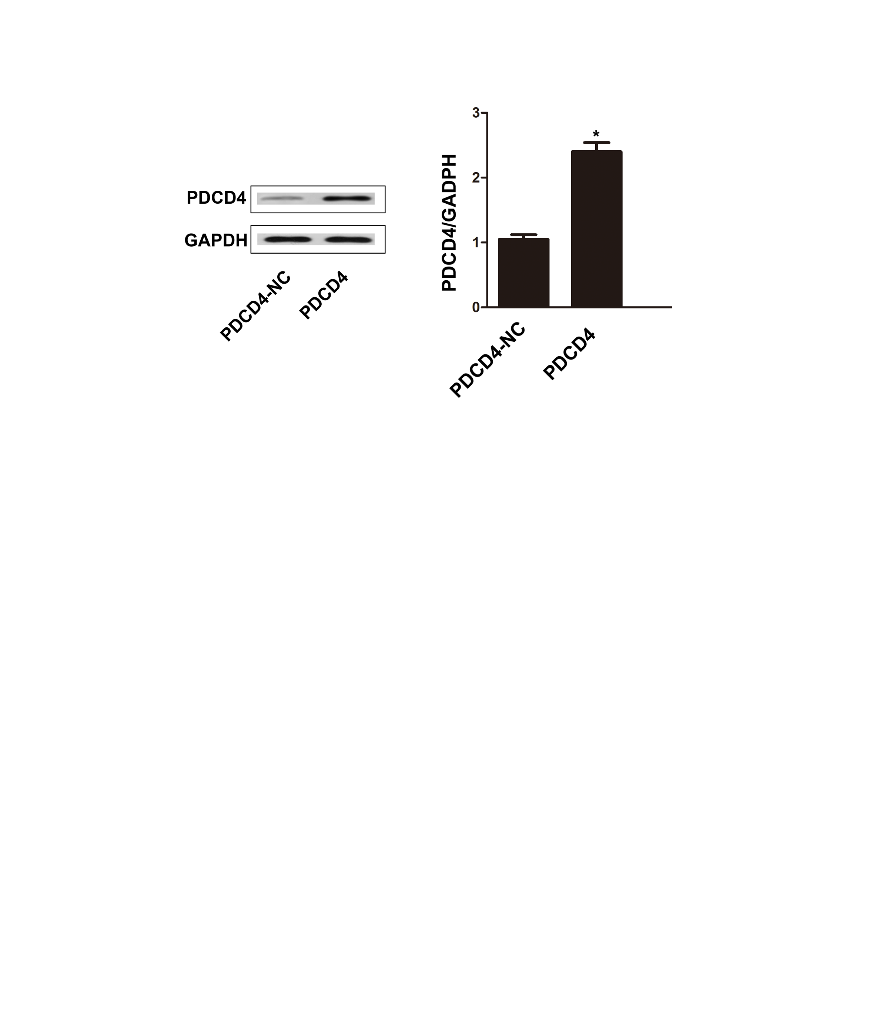


**Supplementary Figure 5. Verification of overexpression PDCD4 in NRVCs by WB.** Expression of PDCD4 protein was detected using western blot assay in NRVCs transfected with PDCD4-overexpression constructs or vehicle control and quantification of PDCD4 normalization to GAPDH (right). **P* < 0.05 compared with PDCD4-sc. Results are representative of three independent experiments.
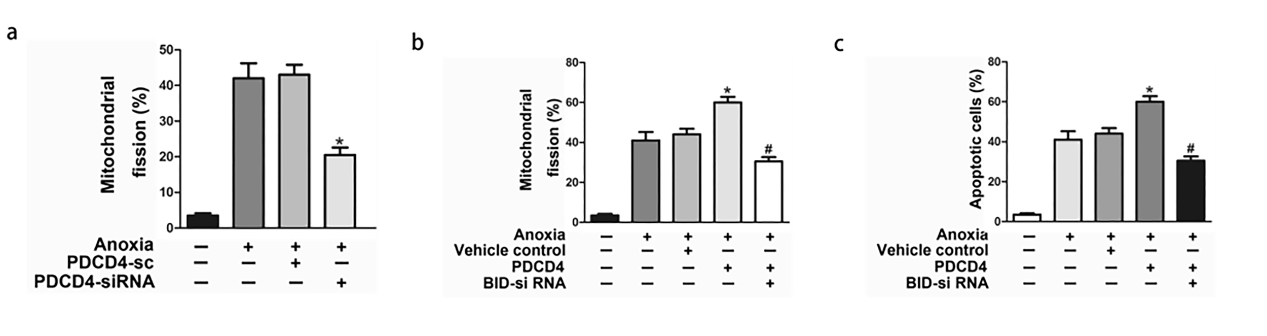


**Supplementary Figure 6. Knockdown of PDCD4 or PDCD4 inhibits mitochondrial fission and apoptosis in NRCV.** (**a**) Knockdown of PDCD4 by PDCD4-siRNA inhibited mitochondrial fission in NRCV induced by anoxia. **P* < 0.05 compared with PDCD4-sc. Results are representative of three independent experiments. (**b**) Overexpression of PDCD4 aggravates mitochondrial fission, but knockdown of BID can prevent mitochondrial fission. Mitochondrial fission in NRVCs transfected with PDCD4-overexpression constructs, BID-si RNA,or vehicle control. **P* < 0.05 compared with vehicle control following anoxia. ^#^*P* < 0.05 compared with PDCD4 following anoxia. Results are representative of three independent experiments. (**c**) Overexpression of PDCD4 aggravates apoptotic cells, but knockdown of BID can attenuate apoptotic cells. Apoptotic cells in NRVCs transfected with PDCD4-overexpression constructs, BID-si RNA,or vehicle control. **P* < 0.05 compared with vehicle control following anoxia. ^#^*P* < 0.05 compared with PDCD4 following anoxia. Results are representative of three independent experiments.


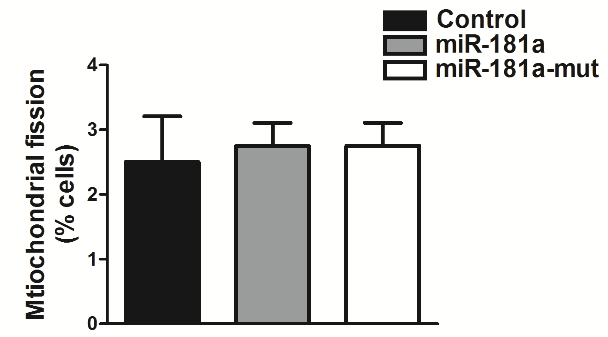


**Supplementary Figure 7. Overexpression of miR-181a alone did not influence the cardiomyocytes mitochondrial fission.** NRVCs were infected with adenoviral miR-181a or miR-181a-mut, and the percentage of cells with mitochondrial fission was determined. Data represent three separate experiments and are expressed as the mean ± SEM.


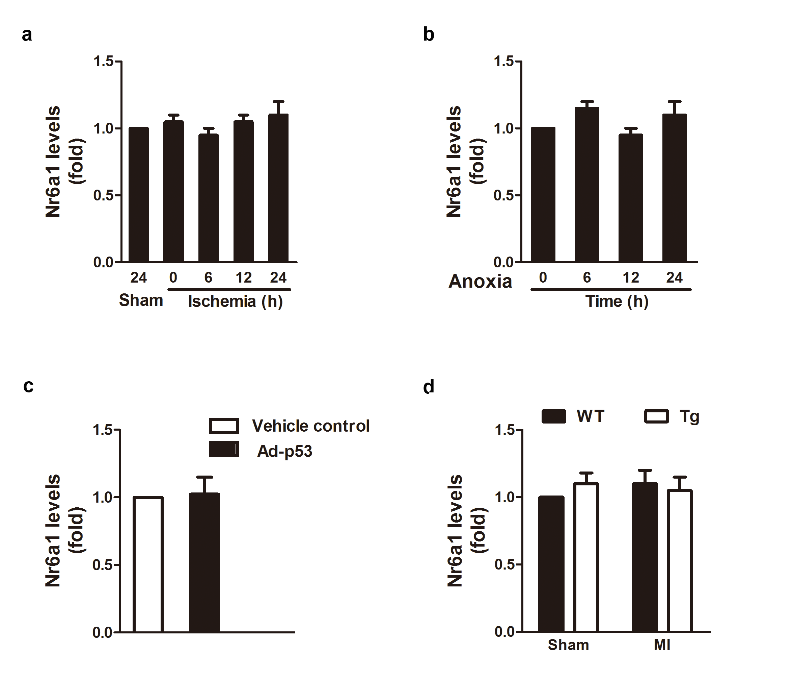


**Supplementary Figure 8. p53 does not significantly alter Nr6a1 expression levels in NRVCs treated with anoxia or not.** (a) Nr6a1 mRNA levels by qRT-PCR analysis in heart of mice from ischemic injury after MI. (b) Nr6a1 mRNA levels determined by qRT-PCR in NRVCs exposed to anoxia. (c) p53 can not significantly Nr6a1 mRNA level. Cardiomyocytes were infected with adenoviral p53 or β-galactosidase (β-gal) and harvested 24 h after infection for detecting Nr6a1 mRNA by qRT-PCR. (d) Nr6a1 mRNA levels in wild-type (WT) and miR-181a transgenic (Tg) mice treated with MI. Nr6a1 mRNA levels were analyzed after 24h MI by qRT-PCR, n = 6. Data represent three separate experiments as mean ± SEM.


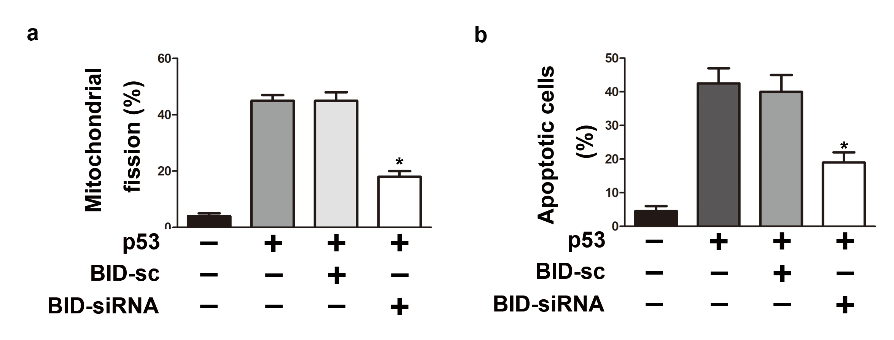


**Supplementary Figure 9. Knockdown of BID attenuates mitochondrial fission and cell apoptosis induced by p53.** NRVCs were infected with adenoviral BID siRNA or the scramble form (BID-sc) and then infected with adenoviral p53. Mitochondrial fission (**a**) and TUNEL assay (**b**) were performed 36 h after p53 infection. **P* < 0.05 compared with p53 + BID-sc.


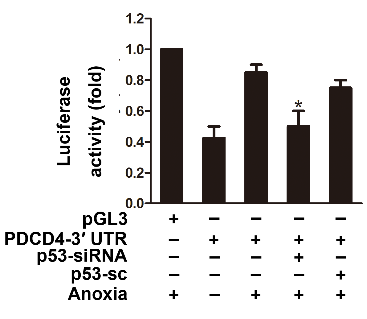


**Supplementary Figure S10. Knockdown of p53 abolishes the translation activity of PDCD4-3′ UTR promoted by anoxia.** NRVCs were infected with the adenoviral p53 siRNA or its scramble form (p53-sc), transfected with the luciferase construct of PDCD4-3′ UTR, and then exposed to anoxia. pGL3 was used as a control. Firefly luciferase activities were normalized to Renilla luciferase activities. Data represent three separate experiments as mean ± SEM **P* < 0.05 conpared with PDCD4-3′ UTR + anoxia.

**Supplementary Methods**

**Cardiac-specific miR-181a transgenic mice generation**

The plasmids were constructed by inserting the miR-181a sequence into the downstream of the cardiac-specific murineα-myosin heavy chain (α-MHC) promoter. Microinjection was performed following standard manipulation. The transgenic mice were genotyped by PCR. The primers for positive transgenic PCR product (278 bp) were as follows: F-primers: GAACCTCAACAAGCTCATGACCA, R-primers: CACACCTCCCCCTGAACCTGAA. The primers for β-actin as internal control PCR product (413 bp) were as follows: F-primers: ACTCCAAGGCCACTTATCACC, R-primers: ATTGTTACCAACTGGGACGACA. The PCR annealing Temp is 60℃.

***In vivo* gene transfer of miR-181a antagomir and adenoviruses harboring p53 siRNA**

A Lipofectamine-mediated in vivo transfection method was used to deliver miR-181a antagomir in an effort to decrease miR-181a levels in the heart of mice as described. With the chest open, 80 ng miR-181a antagomir and 80 ng antagomir control (Shanghai GenePharma Co.) , pretreated with 20 µl lipofectamine 2000 (Invitrogen), were injected through a 29-gauge microinjector into the myocardium (hamilton). Intramuscular injections were made in 4 sites before coronary artery occlusion. Additionally, we injectd 1 × 10^10^ multiplicity of infection of adenoviral p53 siRNA through a 29-gauge microinjector into the myocardium (hamilton) to decrease p53 levels in the heart of mice. After injection, the heart was immediately placed back into the intra-thoracic space followed by manual evacuation of air and was closed with sutures. Experimental measurements were made 24 h after intramuscular injection and myocardial infarction.

Synthesis of miRNAs and sequences of miRNA antagomir

Mouse miR-181a antagomir and antagomir control were synthesized by Shanghai GenePharma Co. The sequence of miR-181a is the exact antisense of the mature miRNA sequence (5′-ACUCACCGACAGCGUUGAAUGUU-3′) and the sequence of antagomir control (5′-UCUACUCUUUCUAGGAGGUUGUGA-3′). miRNA antagomir and antagomir control were stabilized with 2′-O-methyl modification. Rat miR-181a mimic sense sequence is: AACAUUCAACGCUGUCGGUGAGU, miR-181a mimic mutant sense sequence is AACGCCTAACGCUGUCGGUGAGU.

**Quantitative real-time PCR (qRT-PCR)**

miRNA expression in mouse hearts and in culture cardiac cells was determined by quantitative reverse transcriptase–polymerase chain reaction (qRT–PCR). qRT-PCR was performed on cDNA generated from 50 ng of total RNA using the protocol of TaqMan® MicroRNA Assays kit according to the manufacturer’s instructions (Applied Biosystems). Total RNA was extracted from the heart and cells using TRIzol reagent (Invitrogen, Carlsbad, CA, USA) according to the manufacturer’s instructions. qRT-PCR was performed in triplicate using a ABI Prism 7500 sequence detection system (Applied Biosystems). Amplification was performed as follows: 94°C for 5 min and 30 cycles at 94°C for 30 s, 55°C for 30 s, and 72°C for 30 s. The cycle number at which the fluorescent signals of the sample rose above baseline was referred to as Ct (threshold cycle) and was proportional to the target concentration. The miRNA levels were normalized to that of U6 expression. The primers used were as follows: U6, Forward: 5′-GCTTCGGCAGCACATATACTAA-3′, Reverse: 5′-AACGCTTCACGAATTTGCGT-3′; mouse miR-181a, Forward：AACATTCAACGCTGTCG, Reverse：AACTGGTGTCGTGGAG; rat miR-181a, Forward：CCCAATATATGTTAATCTCTTACC, Reverse：TTTTAATAAATTTTTACTTGCTA.

The quantitative detection of col1a1, col3a1, α-SMA and Nr6a1F by qRT-PCR was performed using the TaqMan® MicroRNA Assays kit in an ABI Prism 7500 sequence detection system (Applied Biosystems) according to the manufacturer′s instructions. As an internal control, GAPDH was used for template normalization of the mRNA expression. The primers used were as follows: col1a1, Forward: 5′- GGTCAGACCTGTGTGTTCCC-3′, Reverse: 5′- GGTCCATGTAGGCTACGCTG-3′; Col3a1, Forward: 5′- CAATGTAAAGAAGTCTCTGAAG-3′, Reverse: 5′- CAAACAGGGCCAATGTCCAC-3′; α-SMA, Forward: 5′- CTGTGCTATGTCGCTCTGGA-3′, Reverse: 5′- ATAGGTGGTTTCGTGGATGC-3′;

Nr6a1F, Forward: GTCTACTCCACCACCTATTTTC, Reverse: CCTGTGCTCATTGCCTTGTC; rat GAPDH, Forward: 5′- TGGAGTCTACTGGCGTCTT-3′, Reverse: 5′- TGTCATATTTCTCGTGGTTCA-3′; mouse GAPDH, Forward: 5′- TGTGTCCGTCGTGGATCTGA-3, Reverse: 5′- CCTGCTTCACCACCTTCTTGA-3′.

**Western blot analysis**

Western blotting was performed according to standard protocols. Proteins were extracted and homogenized in a RIPA buffer containing a protease inhibitor cocktail from the whole mouse heart and NRVC washed twice with PBS. The protein concentration was determined using a BCA Protein Assay kit ([Thermo Fisher Scientific](https://www.baidu.com/link?url=AFBbwMef7EEfpz90G26tm8wyglcPQilVyQ1U2KvhF4Bnv67Gy8UuxUyeqdenE0j6O38-1s77zKJWMR8NvoYcmavkIdPwsyvxSbNURQEA_Yy&wd=&eqid=8565b1e700a597b20000000357426983)). Equal number of proteins (30 μg) from each sample was subjected to 12% sodium dodecyl sulfate-polyacrylamide gel electrophoresis (SDS-PAGE) and transferred onto a PVDF membrane (Merck Millipore). The membranes were blocked and incubated with specific primary antibodies (1:1,000) overnight at 4°C, followed by corresponding horseradish peroxidase-conjugated secondary antibodies (Jackson Laboratory). The primary antibody to PDCD4 was from proteintech (12587-1-AP, 52 KD). The primary antibody to BID was from proteintech (10988-1-AP, 22 KD). The primary antibody to p53 was from proteintech (10442-1-AP, 44 KD).As an internal control, membranes were also immunoblotted with an anti-GAPDH antibody (1: 5,000) (10494-1-AP, proteintech, USA). Antigen-antibody complexes were visualized using enhanced chemiluminescence using ECL detection reagents (Abcam) according to the manufacturer’s instructions. The intensities of the protein bands were determined by Quantity One Analysis Software (Bio-Rad). All experiments were repeated three times (technical triplicates), with biological duplicates indicated in each panel.

**Reporter constructions and luciferase assay**

The mutated 3′ UTRs were generated QuikChange II XL Site-Directed Mutagenesis Kit (Stratagene, La Jolla, CA, USA), and mutations (the wild-type PDCD4 3′ UTR: UGUAAG, the mutated PDCD4 3′ UTR: CACGGT) were introduced into the binding sites. The primers for 3′ UTRs of PDCD4 were：The forward primer was: 5′-CCGCTCGAGGCACAGCAACTCTTACAGTCTTAGGTGTT-3′, The reverse primer was: 5′-GGCTCTAGACTAAAGAATCAACAGTGTTTCACATGTTTTATTTTG-3′,; The miR-181a promoter region was amplified from rat genomic DNA to generate wild-type promoter. The forward primer was: 5′-AGGGCCGCCCCGGCCGCACAGTCTATCCCACAGTTCATTAGTT-3′, The reverse primer was: 5′-AGGAATTCGCTGGACTGCTCCTTACCTTGTTGAAATGA-3′. The PCR product was cloned into the vector pGL3 vetor (Promega). To generate reporter vectors bearing miR-181a binding sites, 3′ UTRs of PDCD4 were cloned into the pGL3 vector (Promega) immediately downstream of the stop codon of the luciferase gene. Luciferase activity assay was performed in HEK293T cells using the Dual-Luciferase Reporter Assay System (Promega) according to the manufacturer’s instructions. Luciferase reporter assays were performed in HEK293T cells. HEK293T cells were seeded into 24-well plates and transfected with 200 ng well^–1^ of pGL3-PDCD4-3′ UTR, 400 ng well^–1^ of miR-181a using Lipofectamine 2000 (Invitrogen). Luciferase activity was measured using the Dual-Glo Luciferase Reporter Assay system (Promega) and normalized to Renilla luciferase activity. All assays were performed in triplicate.

To verify p53 bounding to the BS region of miR-181a promoter, luciferase activity measured from NRVCs infected with adenovirus harboring p53 or β-galactosidase (β-gal) and transfected with empty vector (pGL3) or with constructs containing the wild-type (WT) miR-181a promoter or the miR-181a promoter mutated the putative p53 binding sites (m-BS). Firefly luciferase activities were normalized to Renilla luciferase activities.

**siRNA constructions of PDCD4, BID, p53**

The PDCD4 siRNA sense sequence is: 5′-GUCUAAAGGUGGAAAGCGUD-3′; the antisense sequence is: 5′-ACGCUUUCCACCUUUAGACD-3′. The scramble PDCD4 siRNA sense sequence is: 5′-TGGCTTTATGTGTGTGTGG-3′, the antisense sequence is: 5′-CACACCGCAACTACAAACA-3′, BID siRNA sense sequence is: 5′- CCGAAACAAUGACCGUGAU-3′; the antisense sequence is: 5′- AUCACGGUCAUUGUUUCGG-3′. The scramble BID siRNA sense sequence is: 5′-TGTTGCGTATGTTGGTGTG-3′; the scramble antisense sequence is 5′-CACACCAACATACGCAACA-3′. p53 siRNA sense sequence is 5′-CACATGACTGAGGTCGTGA-3′; the antisense sequence is 5′-TCACGACCTCAGTCATGTG-3′. The scramble p53 siRNA sense sequence is 5′-GACGTATGCAGAGTCGTCA-3′; the scramble antisense sequence is 5′-TGACGACTCTGCATACGTC-3′. The siRNA and scrambled siRNA were designed and synthesized by Invitrogen and then verified. They were cloned into p*Silencer* adeno 1.0-CMV vector according to the manufacturer’s instructions.

**Preparations of PDCD4 and p53 overexpression constructs**

PDCD4 and p53 plasmid was synthesized by PCR using mouse genomic DNA as the template. The PDCD4 promoter upstream primer was: 5′-GGGGTACCGAGGCTTGGCTAGTCATG-3′, the PDCD4 promoter downstream primer was: 5′-GAAGATCTGGGCTACAAGAAGGCAG-3′. The p53 promoter upstream primer was: TAGCTAGCATGGAGGAGCCGCAGTCAG; the p53 promoter downstream primer was: TAGGATCCTCAG TCTGAG TCAGGCCCTT. The generated PCR fragment was cloned into the Adeno-XT Expression System (Clontech).
