# Supplementary material for: MiR-181a protects the heart against myocardial infarction by regulating mitochondrial fission via targeting programmed cell death protein 4
Source: Sci Rep. 2024 Mar 19;14:6638. doi: 10.1038/s41598-024-57206-8 (PMC10951332; doi:10.1038/s41598-024-57206-8)
Supplement: Supplementary file 2 — Supplementary Information 2. [file 41598_2024_57206_MOESM2_ESM.pdf]

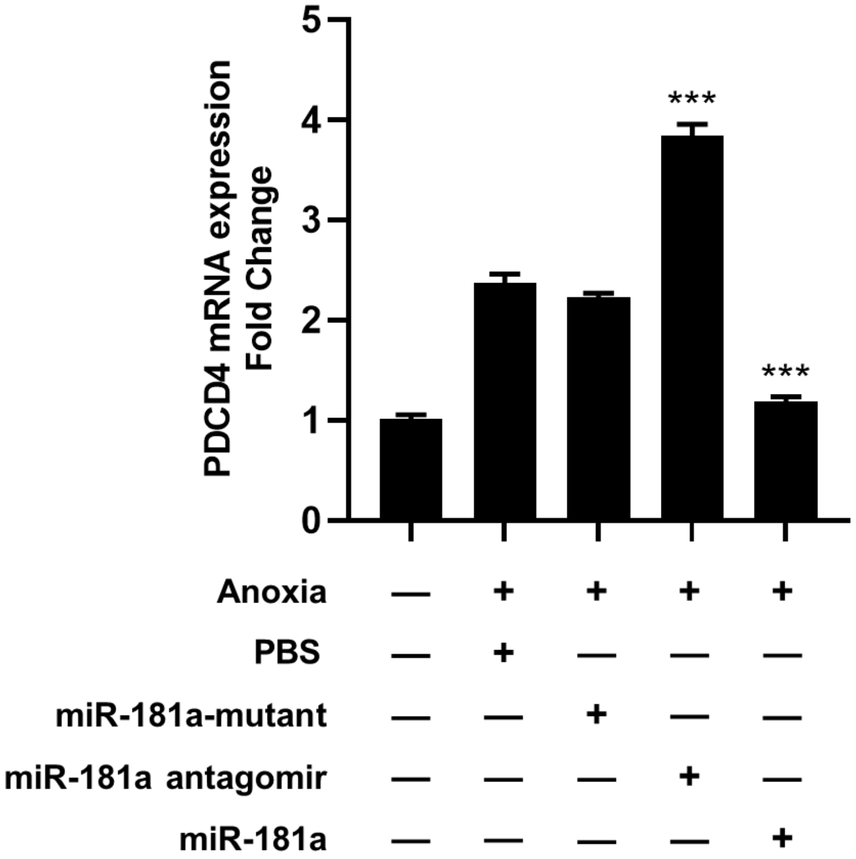

Supplementary Figure 1 for revise. Relative PDCD4 mRNA expression after miR-181a knock down/overexpression. Expression of PDCD4 mRNA was detected using qRT-PCR in NRVCs exposed to anoxia transfected with miR-181a or the miR-181a antagomir and quantification of PDCD4 normalization to GAPDH (right). **P* < 0.05 compared with anoxia alone. Results are representative of three independent experiments.

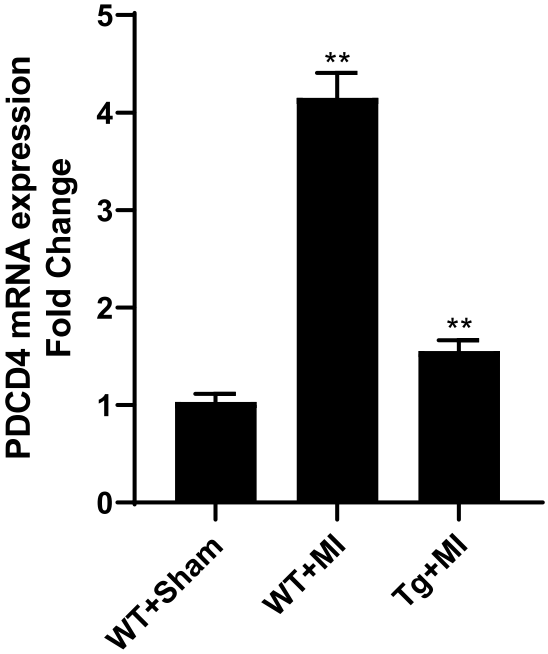

Supplementary Figure 2 for revise. Relative PDCD4 mRNA expression after miR-181a overexpression. miR-181a Tg mice exhibit a low level of PDCD4 mRNA upon MI (n = 6). The expression of PDCD4 mRNA was detected by qRT-PCR.

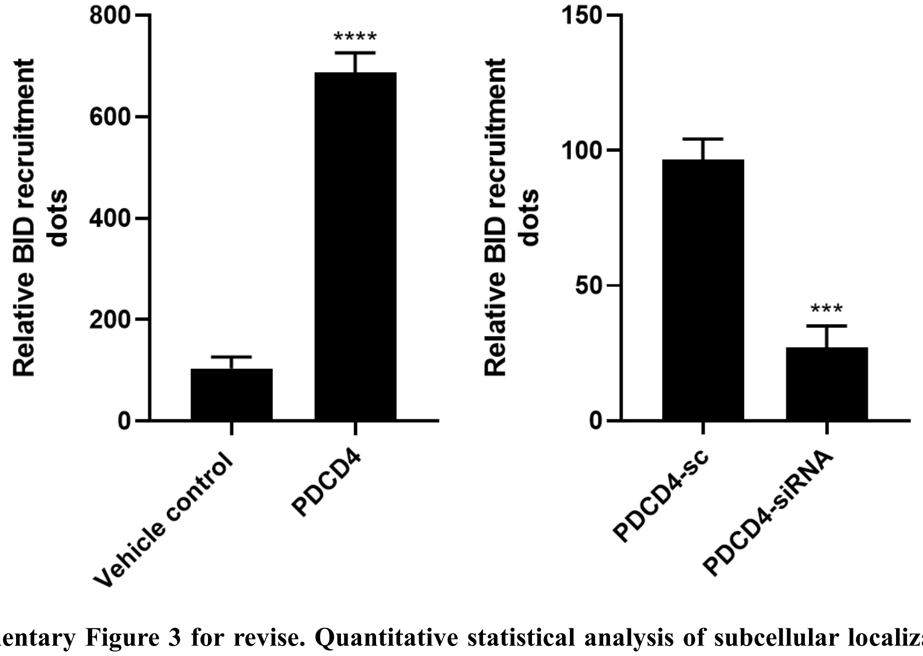

Supplementary Figure 3 for revise. Quantitative statistical analysis of subcellular localization of BID in NRVCs.

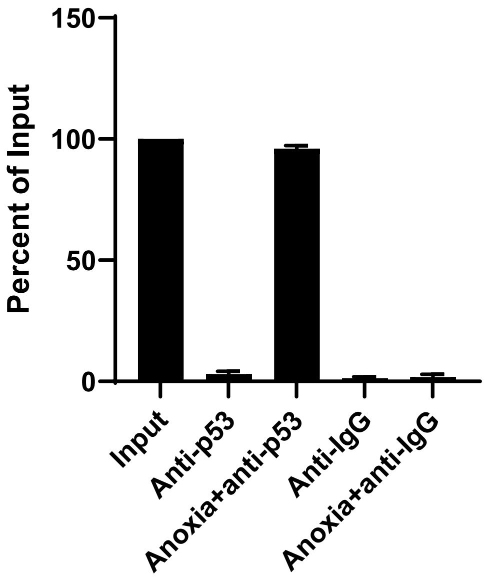

Supplementary Figure 4 for revise. Quantitative statistical analysis of ChIP analysis of p53 binding to the miR-181a promoter in NRVCs treated or not with anoxia.

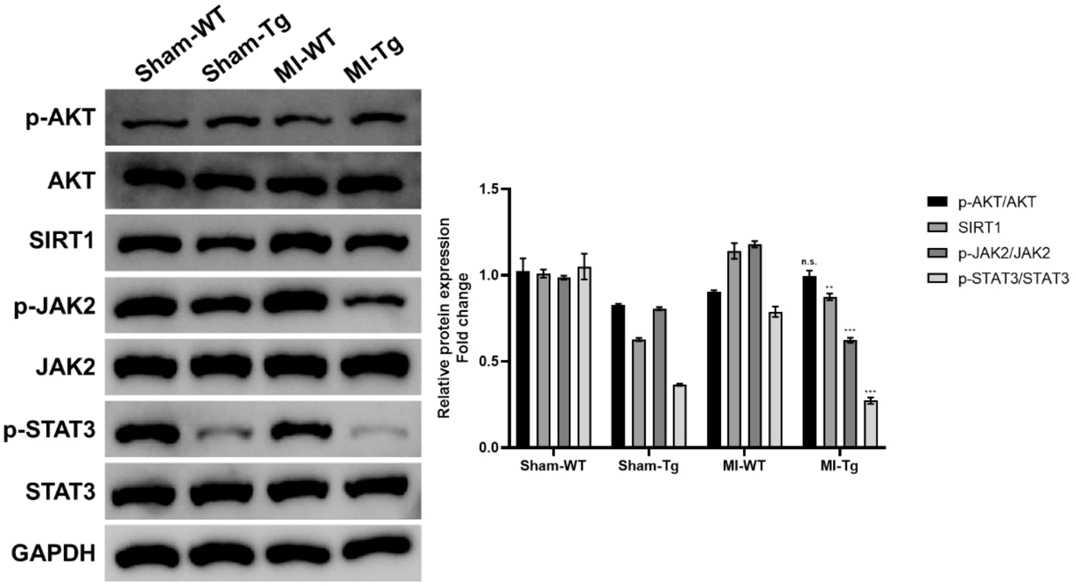

Supplementary Figure 5 for revise. Relative downstream target proteins expression after miR-181a overexpression. Expression of downstream target proteins of miR-181a were detected using western blot assay in WT and Tg mice, and quantification normalization to GAPDH.

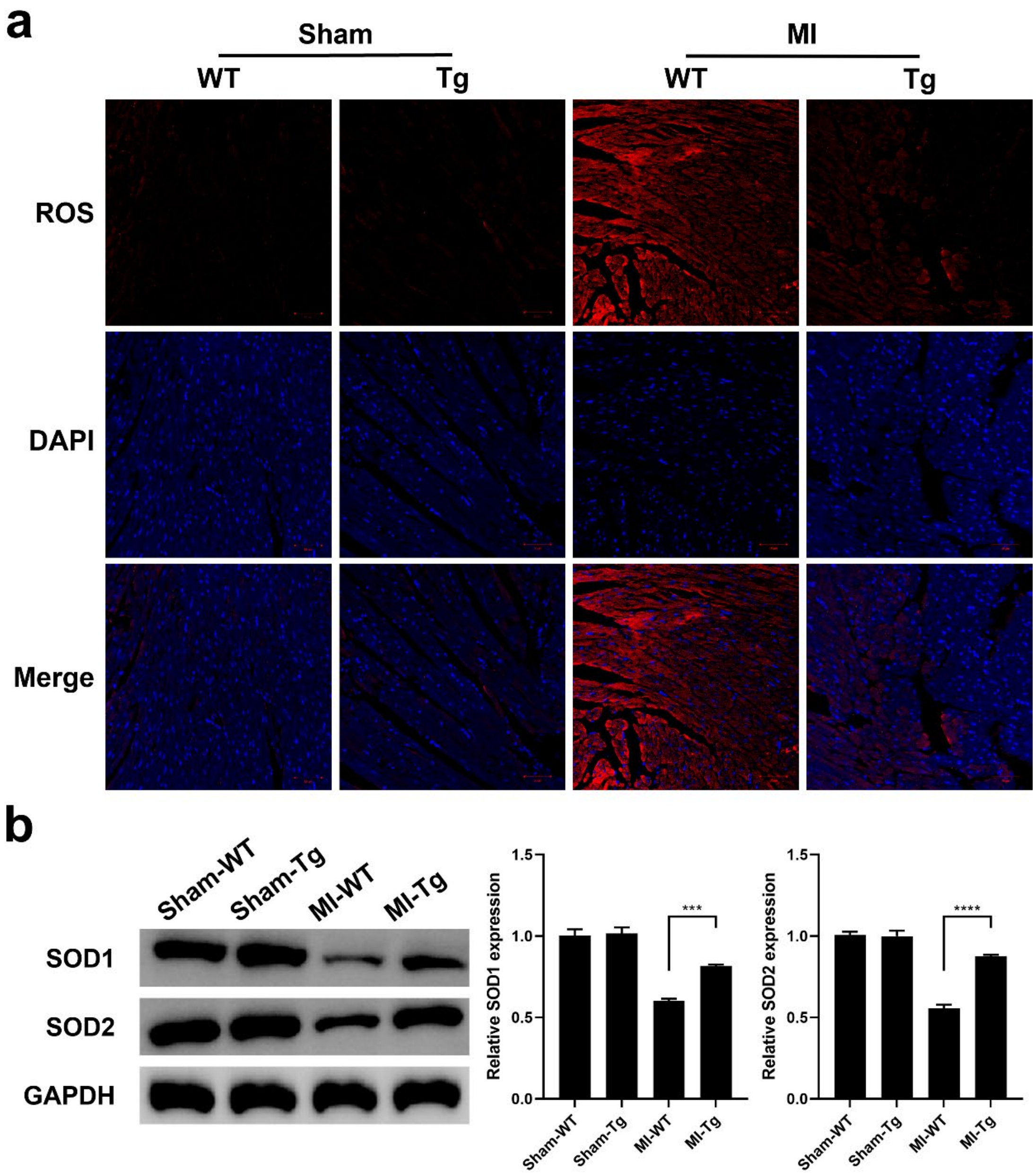

Supplementary Figure 6 for revise. Oxidative stress detection after miR-181a overexpression. (a) Representative ROS images in cardiomyocytes from miR-181a Tg mice or WT mice subjected to MI. **(b)** Expression of relative oxidative stress proteins of miR-181a were detected using western blot assay in WT and Tg mice, and quantification normalization to GAPDH.
