# Supplementary figures and images for: MiR-181a protects the heart against myocardial infarction by regulating mitochondrial fission via targeting programmed cell death protein 4
Source: Sci Rep. 2024 Mar 19;14:6638. doi: 10.1038/s41598-024-57206-8 (PMC10951332; doi:10.1038/s41598-024-57206-8)

3B

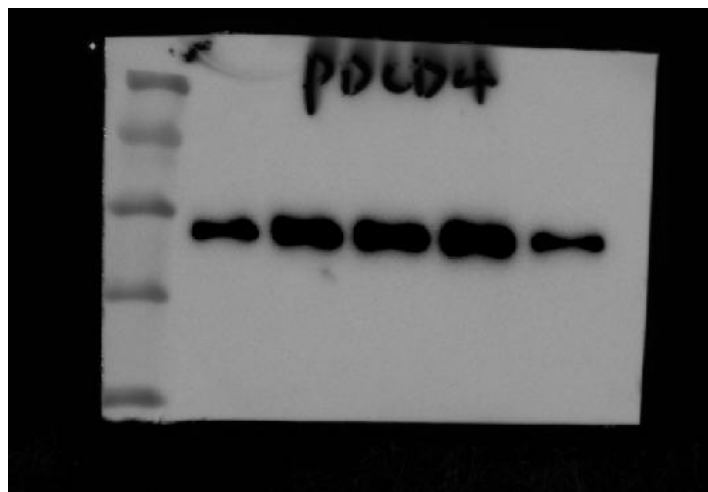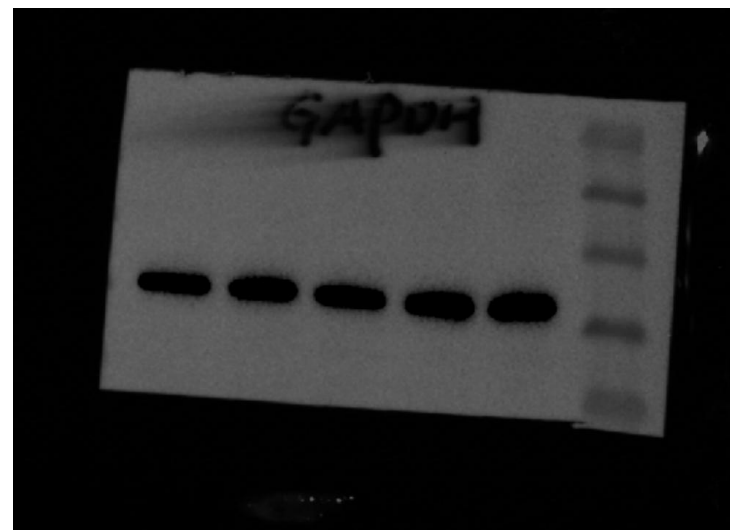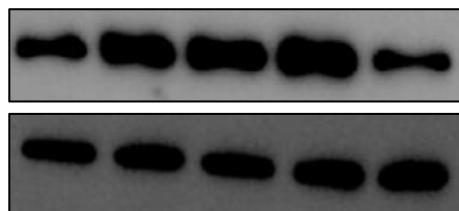

3C

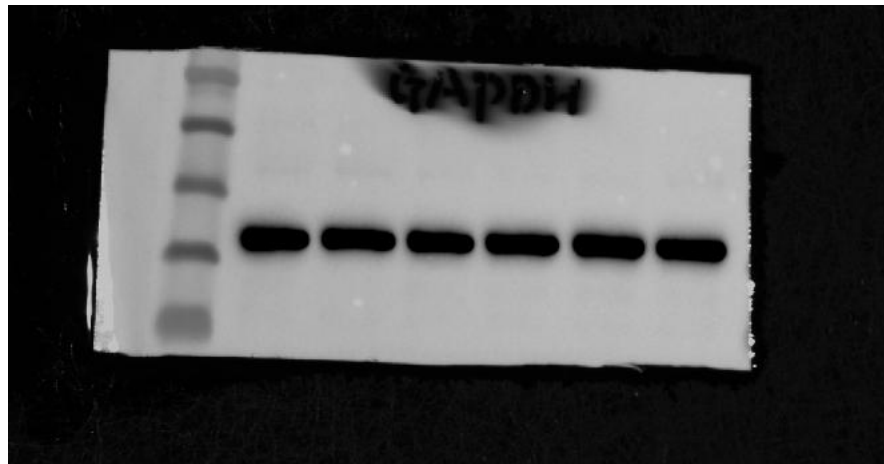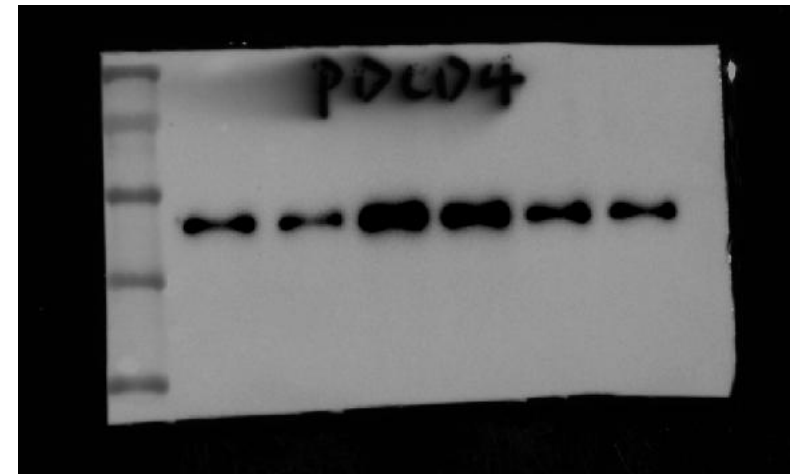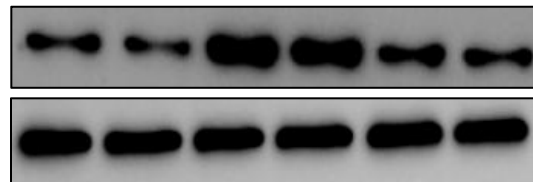

3G

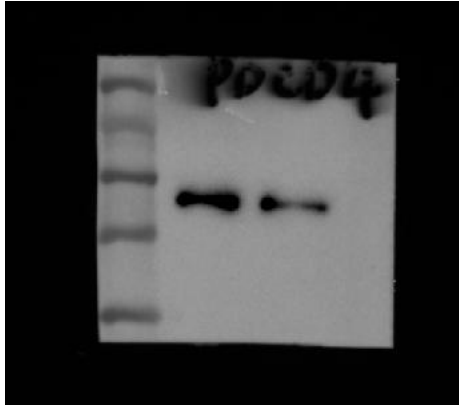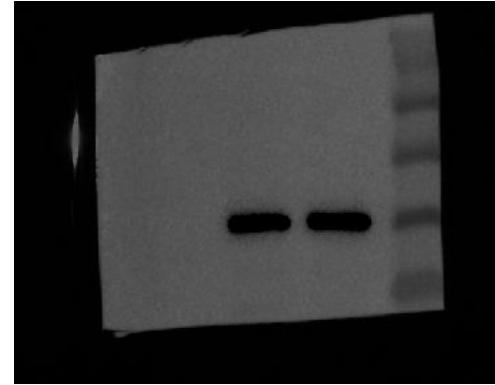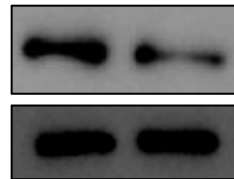

4A

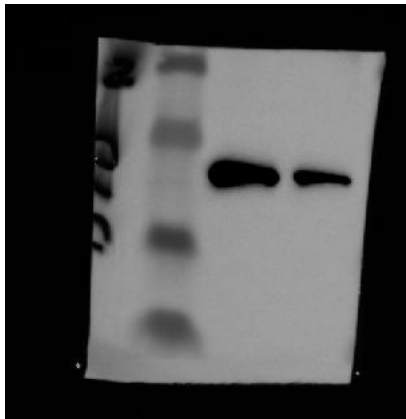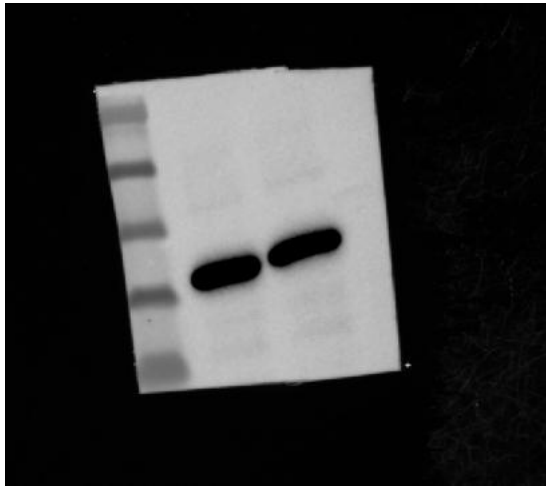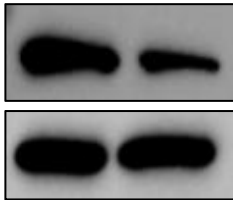

4E

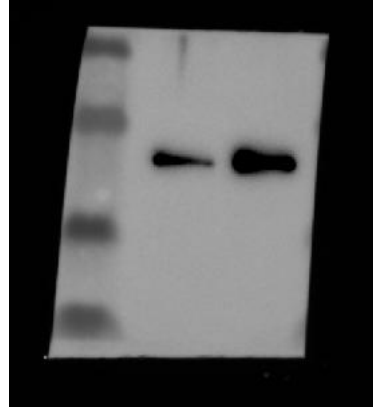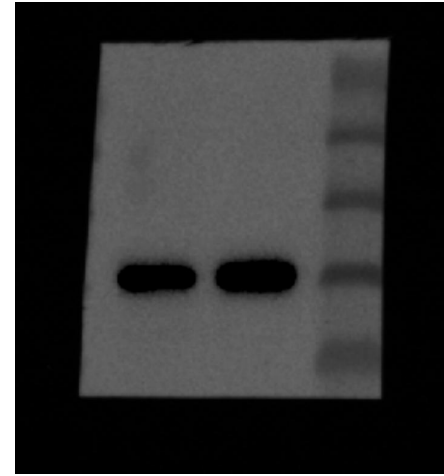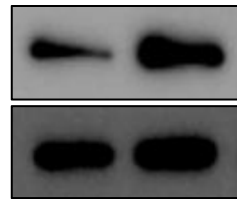

4F

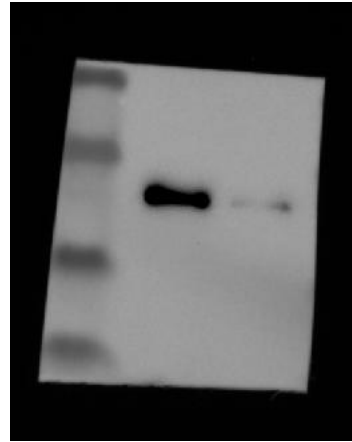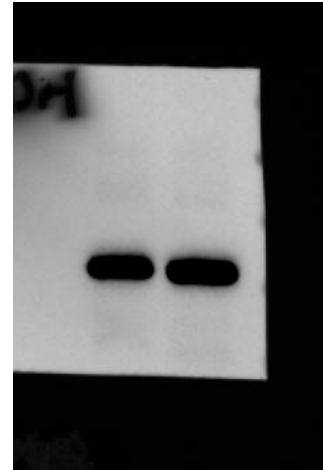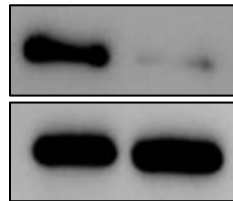

5D

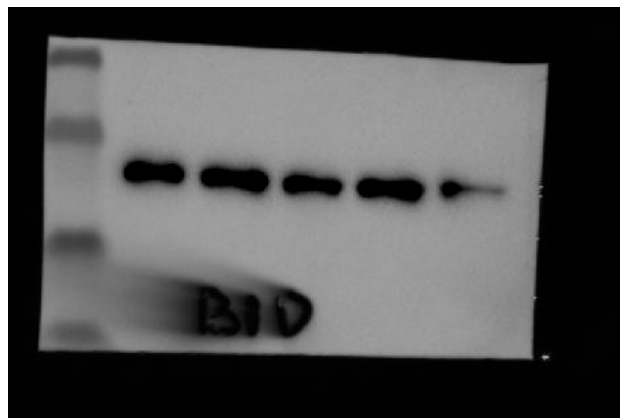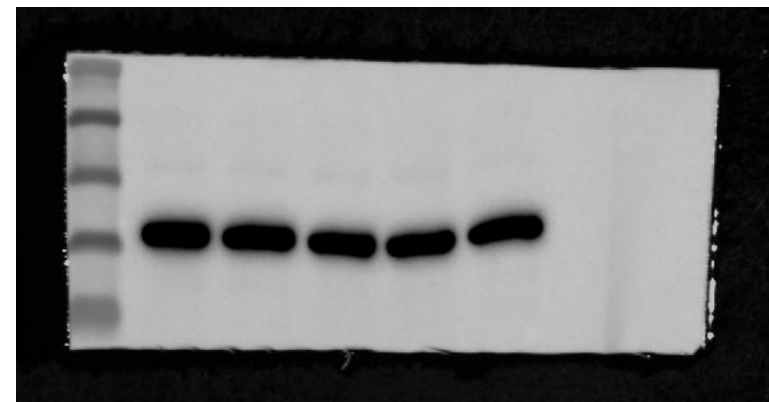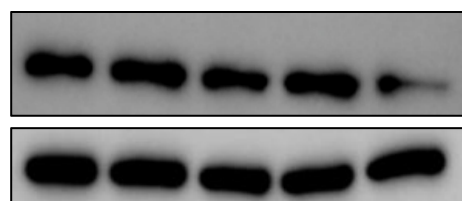

6D

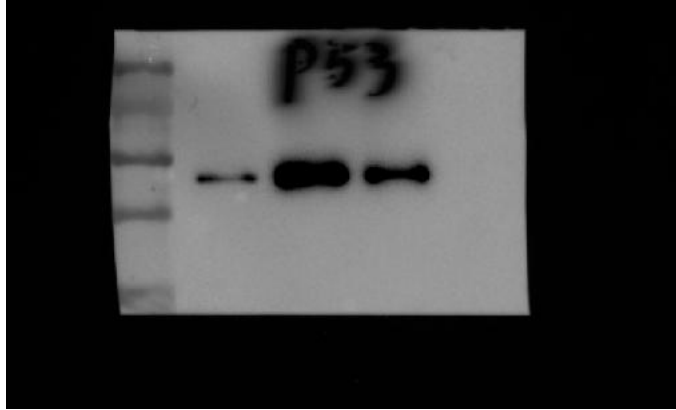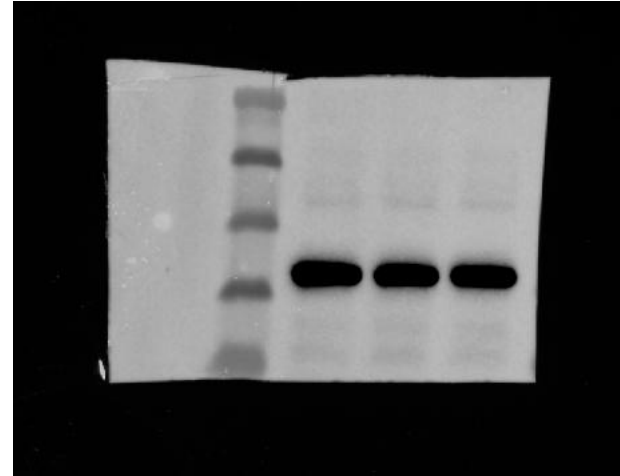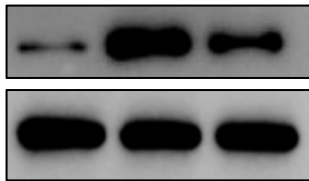

Supplementary Figure 5 for revise

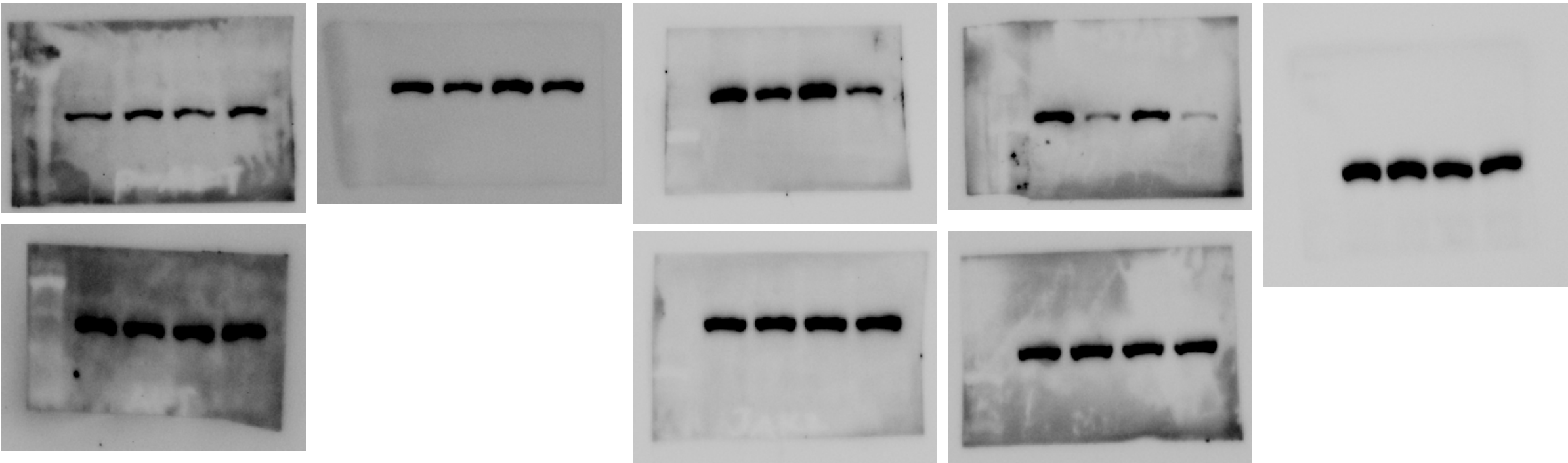

Supplementary Figure 6 for revise

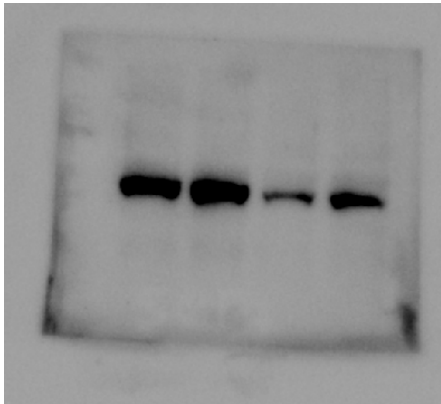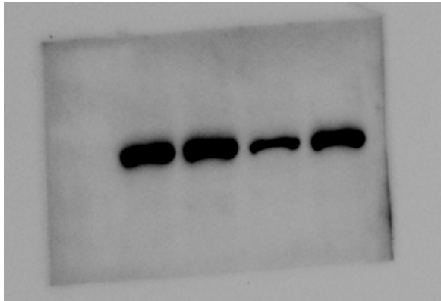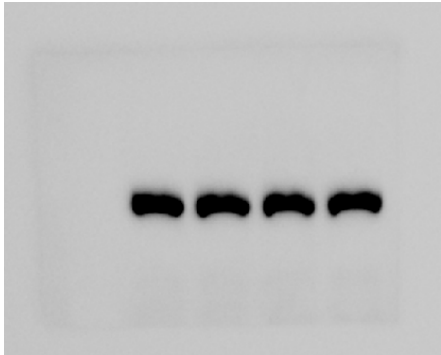

Supplement: Supplementary file 3 — Supplementary Information 3. [file 41598_2024_57206_MOESM3_ESM.pdf]
